# The consequences of sea lamprey parasitism on lake trout energy budgets

**DOI:** 10.1093/conphys/coad006

**Published:** 2023-03-08

**Authors:** Tyler J Firkus, Konstadia Lika, Noah Dean, Cheryl A Murphy

**Affiliations:** Department of Fisheries and Wildlife, Michigan State University, 480 Wilson Road, East Lansing, MI 48824, USA; Department of Biology, University of Crete, GR-70013,P.O.Box 2208, Heraklion, Crete, Greece; Department of Fisheries and Wildlife, Michigan State University, 480 Wilson Road, East Lansing, MI 48824, USA; Department of Fisheries and Wildlife, Michigan State University, 480 Wilson Road, East Lansing, MI 48824, USA

## Abstract

Parasitism is an energetically costly event for host species. Dynamic energy budget (DEB) theory describes the metabolic dynamics of an individual organism through its lifetime. Models derived from DEB theory specify how an organism converts food to reserves (maintenance-free energy available for metabolism) and allocates mobilized reserves to maintenance, growth (increase of structural body mass) and maturation or reproduction. DEB models thus provide a useful approach to describe the consequences of parasitism for host species. We developed a DEB model for siscowet lake trout and modeled the impact of sea lamprey parasitism on growth and reproduction using data collected from studies documenting the long-term effects following a non-lethal sea lamprey attack. The model was parameterized to reflect the changes in allocation of energy towards growth and reproduction observed in lake trout following sea lamprey parasitism and includes an estradiol module that describes the conversion of reproductive reserves to ovarian mass based on estradiol concentration. In our DEB model, parasitism increased somatic and maturity maintenance costs, reduced estradiol and decreased the estradiol-mediated conversion efficiency of reproductive reserves to ovarian mass. Muscle lipid composition of lake trout influenced energy mobilization from the reserve (efficiency of converting reserves allocated to reproduction into eggs) and reproductive efficiency. These model changes accurately reflect observed empirical changes to ovarian mass and growth. This model provides a plausible explanation of the energetic mechanisms that lead to skipped spawning following sea lamprey parasitism and could be used in population models to explore sublethal impacts of sea lamprey parasitism and other stressors on population dynamics.

## Teaser

We developed a dynamic energy budget model that describes energy allocation towards growth, maturation, and reproduction for the siscowet lake charr (Salvelinus namaycush). Using this model, we simulate changes in energy allocation observed following parasitism from sea lamprey (Petromyzon marinus), a significant stressor for lake charr in the Laurentian Great Lakes.

## Introduction

One of the most important stressors for lake trout (*Salvelinus namaycush*) in the Laurentian Great Lakes is parasitism from non-native sea lamprey (*Petromyzon marinus*). Sea lamprey are large ectoparasites that feed by attaching to host fish with a suction-cup-like mouth, mechanically removing scales and tissue with a rasping tongue and consuming blood and tissue (Lennon, 1954). Lake trout are the preferred host species for sea lamprey in the Laurentian Great Lakes (Harvey *et al.,* 2008; Johnson *et al.,* 2021). Following a sea lamprey attack, hosts face a series of complications including osmotic imbalances from a large open wound (Ebener *et al.,* 2006; Goetz *et al.,* 2016; Firkus *et al.,* 2020), low hematocrit from loss of blood (Edsall and Swink, 2001) and introduced compounds from sea lamprey buccal gland secretions (Goetz *et al.,* 2016). Sea lamprey parasitism is often lethal to lake trout (Swink, 1990, 2003; Madenjian *et al.,* 2008), but hosts that survive are faced with energetic deficits and alterations to reproductive and growth physiology, often leading to a complete cessation of spawning (Goetz *et al.,* 2016; Smith *et al.,* 2016; Firkus *et al.,* 2022). Accordingly, when sea lamprey were introduced to the Laurentian Great Lakes in the late 1800s following construction of the Welland Canal, lake trout populations sharply declined (Hansen, 1999; Muir *et al.,* 2013).

Understanding the sublethal effects of sea lamprey parasitism on host lake trout physiology is critical for evaluating the effects on lake trout populations. Empirical measurements of sublethal effects at the molecular, cellular, or tissue level of biological organization provide important information but are not sufficient to understand effects on individual fish performance. One valuable tool for modeling the energetic consequences of stressors at lower levels of biological organization and linking them to individual effects is dynamic energy budget (DEB) theory (Kooijman, 2010; Martin *et al.,* 2013). DEB theory provides a modeling framework based on thermodynamic principles that describe the metabolic dynamics and energy partitioning of an individual organism through its entire life cycle (Kooijman, 2010; Sousa *et al.,* 2010; Jusup *et al.,* 2017). DEB models are adaptable and can be developed for any species. Model parameters are estimated from observed physiological data from a given species. Once parameterized, a DEB model can describe energy dynamics and simulate growth, reproduction and life history characteristics under different environmental conditions, such as temperature and feeding regimes, and stressors, including contaminants, disease and parasitism at any point in an organism’s life cycle (Kooijman, 2010). Because these models consider the whole organism and can simultaneously account for stress acting on multiple physiological functions, they are well suited to assimilating empirically measured sublethal effects of sea lamprey parasitism on lake trout to help understand the consequences for the entire organism. DEB models can also be modified to account for and integrate multiple sub-organismal processes to better explain energy dynamics. This study is a novel application of a DEB model to predict impact of sea lamprey parasitism on host fish; specifically the model focuses on alterations to reproduction and growth and accounts for variation in estradiol concentrations and muscle lipid concentrations.

We parameterized a DEB model for female siscowet lake trout using available life history data from the literature and used the resulting model to explore the effects of sea lamprey parasitism on reproduction, growth and other life history characteristics. Lake trout display tremendous variation throughout their range; four currently recognized lake trout ecomorphs are present in Lake Superior alone, differing in morphology, habitat preference, metabolism and life history characteristics (Moore and Bronte, 2001; Goetz *et al.,* 2014; Muir *et al.,* 2014, 2016; Sitar *et al.,* 2020). A general lake trout DEB model was developed using data from an inland strain of lake trout (Kooijman, 2019), but because the metabolic dynamics and response to sea lamprey parasitism differ so dramatically for the siscowet ecomorph (Goetz *et al.,* 2010, 2014, 2016; Smith *et al.,* 2016; Sitar *et al.,* 2020; Firkus *et al.,* 2022), it was necessary to develop a separate siscowet-specific model. The use of empirical data from siscowet lake trout to inform our DEB model provides a more accurate framework to explore the influence of sea lamprey parasitism as siscowets have the highest rates of observed sea lamprey wounding in the Laurentian Great Lakes (Horns *et al.,* 2003; Sitar *et al.,* 2008). We focused on female siscowet lake trout because fecundity estimates are more important for informing population models in the future, and there is relatively little information available for siscowet milt concentrations.

In prior studies, we empirically measured the influences of sea lamprey parasitism on siscowet lake trout growth, reproduction, energy storage and gene expression (Goetz *et al.,* 2016; Smith *et al.,* 2016; Firkus *et al.,* 2022) which provide critical information for accounting for the effects of sea lamprey parasitism in DEB. For female siscowet lake trout that survive sea lamprey parasitism, a common outcome is skipped spawning whereby an individual forgoes reproductive output completely and instead allocates energy towards surviving the stress associated with the parasitism event. In addition to parasitism, energy storage in the form of muscle lipids and plasma estradiol concentrations also play an important role in the reproductive success of siscowet lake trout and the likelihood of skipping spawning (Sitar *et al.,* 2014; Goetz *et al.,* 2017; Firkus *et al.,* 2022). Sea lamprey parasitism dramatically increases the likelihood of skipping spawning for an individual, but if the lake trout has high muscle lipid and plasma estradiol concentrations, the negative consequences of parasitism can be overcome. Conversely, if muscle lipid and plasma estradiol concentrations are low, the lake trout is likely to skip spawning even in the absence of parasitism. These empirically measured effects can be used to inform how different DEB parameters are stressed under parasitism and allow alterations to reproductive output, growth and energy storage to be estimated within the context of the whole lake trout energy budget. Once the effects of parasitism are modeled appropriately, they can be used to explore the effects of parasitism under a variety of scenarios and ultimately inform stock-recruitment relationships, individual-based models and other tools critical for the management of lake trout in the Laurentian Great Lakes in efforts to restore naturally reproducing populations.

**Figure 1:**
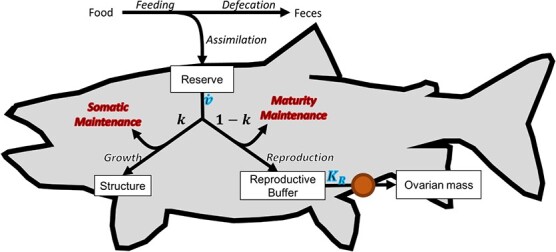
General overview of the structure of the DEB model. Red highlights indicate the components of the model that were altered to simulate the effects of parasitism and blue highlights indicate components altered due to muscle lipid concentration. The orange circle between reproductive buffer and ovarian mass represents the egg sub-model that allows differences in plasma estradiol concentration to influence ovarian mass synthesis.

## Methods

### General model description

To explore the influence of sea lamprey parasitism on siscowet lake trout reproduction and growth, we first developed a base DEB model that described the energy allocation and dynamics of siscowet lake trout throughout their entire lifecycle that accounts for energetic tradeoffs constrained by life history. The general structure, equations and assumptions of DEB models have been thoroughly covered previously (Sousa *et al.,* 2008, 2010; Kooijman, 2010; Jusup *et al.,* 2017). Briefly, DEB models are described by four state variables (reserve energy, structural mass, cumulative energy invested to maturation for juveniles and energy invested in reproduction for adults), and a set of differential equations and model parameters dictate energy flux to each compartment (Kooijman, 2010) (Figure 1). Energy enters an organism through uptake of food (with a fraction removed as feces) and enters a reserve pool. In DEB models, reserve represents all tissue that does not require energy for maintenance and is readily metabolizable as a source of usable energy (Jusup *et al.,* 2017). Energy is then mobilized from the reserve at a given rate and allocated towards somatic functions and maturation/reproduction following the κ-rule. The κ-rule states that a fixed portion (κ) of mobilized energy is allocated towards somatic maintenance (e.g. maintenance of existing structural mass, mean level of movement costs and production of scales) and growth (increase in structural mass), while the remaining fraction (1-κ) is allocated towards maturity maintenance and maturation (for juveniles) or reproduction (for adults). Maturation involves continuous energy investment as the organism becomes more complex and prepares the body for the mature adult state. For example, the preparation of reproductive machinery and development of immune defense systems require more energy as an organism matures (Kooijman, 2010). Maturity maintenance is the energy spent to maintain the current state of complexity. DEB models handle maturity by tracking the cumulative investment of energy towards maturation, and once a specified threshold is reached (called puberty), mobilized energy is then allocated towards a reproductive buffer for later allocation to reproductive activities, such as egg production (Kooijman, 2010; Jusup *et al.,* 2017). Somatic and maturity maintenance processes (e.g. protein turnover, activity, immune function, metabolizing and excreting toxicants, etc.) have priority and are paid first before remaining energy can be allocated to growth or the reproduction buffer. A portion of the energy allocated to reproduction matures to ripe reproductive matter (hereafter referred to as ovarian mass; Kooijman, 2010). Table 1 summarizes the state variables and their dynamics, and a generalized overview of energy allocation processes is shown in Figure 1.

**Table 1:** State variables, mass fluxes and dynamics of the standard DEB model including the egg module. Parameters are defined in Tables 2 and 3.

*State variables*	*unit*	*Description*
${M}_V$	mol	Structural mass
$L$	cm	Structural volumetric length: ${\left({M}_V/\left[{M}_V\right]\right)}^{1/3}$
${M}_E$	mol	Mass of reserve
${m}_E$	mol mol^−1^	Reserve density: ${M}_E/{M}_V$
${M}_H$	mol	mass investment into maturation
${M}_R,{M}_{OV}$	mol	mass investment to reproduction (unripe, ripe)
		
*Fluxes* (mol/d)		
${\dot{J}}_{EA}$	Assimilation rate: $\left\{{\dot{J}}_{EAm}\right\}f{L}^2$	
${\dot{J}}_{EC}$	Reserve mobilization rate: ${M}_{\mathrm{E}}\Big(\frac{\dot{v}}{L}-\dot{r\Big)}$ with$\dot{r}=\frac{{\dot{j}}_{EAm}{\mathrm{m}}_{\mathrm{E}}/{\mathrm{m}}_{\mathrm{E}\mathrm{m}}-{\dot{j}}_{EM}/\mathrm{\kappa}}{{\mathrm{m}}_{\mathrm{E}}+{\mathrm{y}}_{\mathrm{E}\mathrm{V}}/\mathrm{\kappa}}$ if $\frac{{\dot{j}}_{EAm}{\mathrm{m}}_{\mathrm{E}}}{{\mathrm{m}}_{\mathrm{E}\mathrm{m}}}\ge \frac{{\dot{j}}_{EM}}{\mathrm{\kappa}}$^(*)^$\dot{r}=0$ if $\frac{{\dot{j}}_{EAm}{\mathrm{m}}_{\mathrm{E}}}{{\mathrm{m}}_{\mathrm{E}\mathrm{m}}}<{\dot{j}}_{EM}/\mathrm{\kappa}$ and ${M}_R\ \mathrm{or}\ {M}_{OV}>0$$\dot{r}=\frac{{\dot{j}}_{EAm}{\mathrm{m}}_{\mathrm{E}}/{\mathrm{m}}_{\mathrm{E}\mathrm{m}}-{\dot{j}}_{EM}/\mathrm{\kappa}}{{\mathrm{m}}_{\mathrm{E}}+{\kappa}_G{\mathrm{y}}_{\mathrm{E}\mathrm{V}}/\mathrm{\kappa}}$ if $\frac{{\dot{j}}_{EAm}{\mathrm{m}}_{\mathrm{E}}}{{\mathrm{m}}_{\mathrm{E}\mathrm{m}}}<{\dot{j}}_{EM}/\mathrm{\kappa}$ and ${M}_R\ \mathrm{and}\ {M}_{OV}=0$${\dot{j}}_{EAm}=\left\{{\dot{J}}_{EAm}\right\}{M}_V^{-1/3}{\left[{M}_V\right]}^{-2/3}$	
${\dot{J}}_{EM}$	Somatic maintenance rate: ${j}_{EM}{L}^3$	
${\dot{J}}_{EJ}$	Maturity maintenance rate: ${\dot{k}}_{\mathrm{J}}\min \big\{{M}_H,{M}_H^p\big\}$	
${\dot{J}}_{ER}$	Energy flux to maturation/reproduction: $\left(1-\kappa \right){\dot{J}}_{EC}-{\dot{J}}_{EJ}$	
${\dot{J}}_{OV}$	Energy flux to ovaries formation: ${b}_H\frac{M_H}{M_V}\frac{M_R}{M_V}{M}_V$	
*Dynamics*		
$\frac{d}{dt}{M}_V$ = $\dot{\text{r}}{M}_V$$\frac{d}{dt}{M}_E={\dot{J}}_{EA}-{\dot{J}}_{EC}$$\frac{d}{dt}{M}_H=\big({M}_H<{M}_H^p\big)\ {\dot{J}}_{ER}$$\frac{d}{dt}{M}_R=\big({M}_H={M}_H^p\big)\left(\ {\dot{J}}_{ER}-{\dot{J}}_{OV}\right)$^(**)^$\frac{d}{dt}{M}_{OV}={\kappa}_R{\dot{J}}_{OV}$^(**)^		
^(*)^_Condition to meet somatic maintenance costs_ ^(**)^_Modified to cover maintenance costs_		

The standard (std) DEB model is the simplest model in the family of DEB models which can be adapted to model most species (Marques *et al.,* 2018). Because DEB models are adaptable to any species, they use terminology that attempts to be species generic. We used the abj typified DEB model that accounts for metabolic acceleration, a DEB term that refers to rapid growth during early development, following initiation of exogenous feeding (generalized as birth in DEB terminology) (Kooijman, 2014; Lika *et al.,* 2014). Metabolic acceleration occurs well before the maturity threshold for puberty and might or might not coincide with metamorphosis (a DEB term that refers to rapid change in morphology). Although lake trout do not undergo metamorphosis, they do undergo rapid growth post-hatch making the abj model appropriate. The abj DEB model differs from the std DEB model by allowing for the rapid increase in respiration and change in body shape that occurs during the larval or post-hatch stages of most fish species and includes one additional parameter, the maturity threshold at metamorphosis The abj model has been used for many actinopterygians (Lika *et al.,* 2022).

Because we are exploring the effects of parasitism, a substantial stressor that potentially affects maintenance costs, we also implemented rules that describe energy use when available energy in the κ fraction is not sufficient to meet somatic maintenance demands (see Table 1). If there is insufficient energy available to meet somatic maintenance requirements, growth ceases and maintenance costs are paid from the energy available for reproductive functions (i.e. reproductive buffer and/or ovarian mass in proportion to their availability). If there is insufficient energy available in the κ fraction, ovarian mass and the reproductive buffer, energy is then taken from structure and the organism loses structural mass or “shrinks” (Augustine *et al.,* 2014).

### Egg module

To model our observed reproductive processes from Firkus *et al.* (2022) in our lake trout DEB, we added an egg module that allows reproductive hormone dynamics to dictate the conversion of energy in the reproductive buffer into eggs. Previous laboratory studies suggest that estradiol concentration modulates the effects of sea lamprey parasitism on lake trout reproduction (Smith *et al.,* 2016; Firkus *et al.,* 2022), so it is necessary to account for estradiol's role in our model. A similar approach to incorporating hormone dynamics into a DEB model is outlined in Murphy *et al*. (2018) and Muller *et al.* (2019); however, we simplified this approach so that estradiol concentration was the only required input. This approach more explicitly describes the processes involved in egg development and allowed the model to account for differences in estradiol concentration in parasitized and unparasitized individuals. In the egg module, reproductive reserve (energy available for use towards reproduction) molecules are combined with estradiol to synthesize the egg yolk protein vitellogenin. Processes that take place in the blood plasma volume or liver are taken proportional to the structural mass. The energy flux for egg mass production is triggered by estradiol density of (i.e. the ratio of mass of estradiol in plasma and the structural mass, ${m}_{E2}=\frac{M_{E2}}{M_V}$ and follows the law of mass action with the reproductive reserve density (${m}_R=\frac{M_R}{M_V}$). Vitellogenin production occurs in the liver and is secreted into plasma and travels to the ovaries where it is absorbed by ovarian follicles; all processes involved are proportional to the structural mass of the fish ${M}_V$. Thus, the rate of egg mass production is given by ${\dot{J}}_{OV}={b}_H{m}_{E2}{m}_R{M}_V$, where the parameters describing the conversion of reserve, estradiol and vitellogenin to egg mass are absorbed in the proportionality constant ${b}_H$. The dynamics of the egg ovarian mass, ${M}_{OV}$, are given in Table 1.

**Figure 2:**
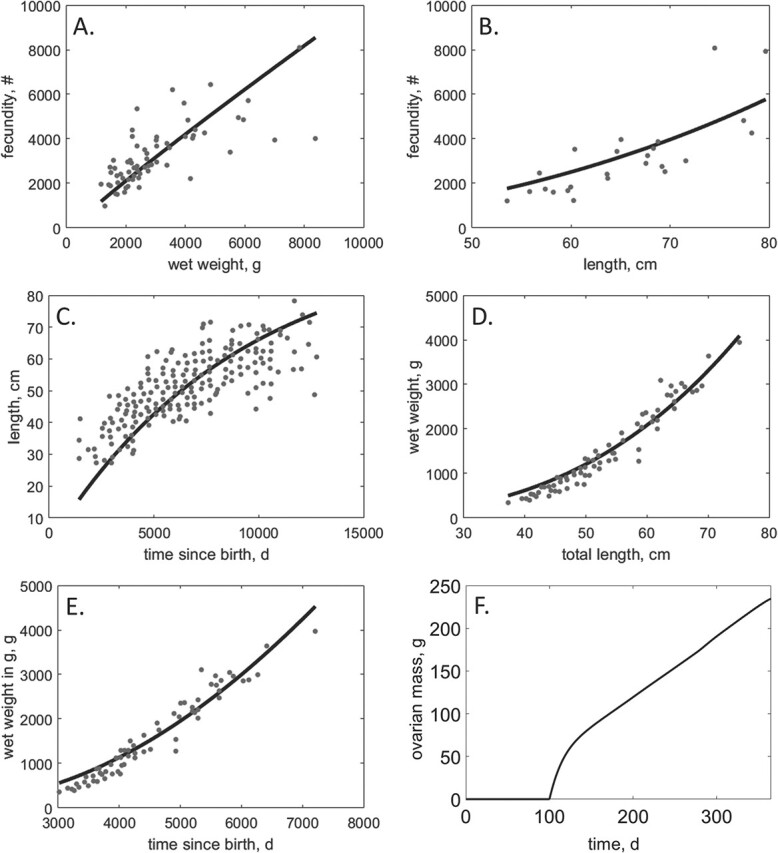
DEB model predictions (black line) compared to univariate data provided from the literature (grey dots). Fecundity as a function of wet weight (A), length (B), length at time (C), wet weight at time (time since exogenous feeding; D), wet weight–total length (E) and ovarian mass at time (time in 1 year prior to reproduction; F).

Data for estradiol were obtained from laboratory studies of siscowet lake trout (Firkus *et al.,* 2022) as ng/ml of plasma. These data were linked to the model variable that accounts for the mass of estradiol ${M}_{E2}$ (in C-mol): ${M}_H={10}^{-9}\left[{E}_2\right]{V}_{pl}/{w}_H$, where $\left[{E}_2\right]$ is the estradiol concentration (ng/ml of plasma), ${w}_H$ is the molecular weight of estradiol (15.1 g/C-mol) and ${V}_{pl}$ is the total volume of plasma in a lake trout in ml given by the following equation: ${V}_{pl}={\beta}_{pl}{W}_w/100$, where ${W}_w$ is wet weight and ${\beta}_{pl}$ is the proportionality constant (averaged value of 2.86% from Gingerich *et al.,* 1987 and Gingerich and Pityer, 1989). The total wet weight ${W}_w$ has contributions from structural mass, reserve mass and ripe (${M}_{OV})$ and unripe (${M}_R)$r reproductive mass: ${W}_w=\frac{w_V{M}_V}{d_V}+\frac{w_E\left({M}_E+{M}_R+{M}_{OV}\right)}{d_E}$, where ${w}_V,{w}_E$ and ${d}_V,{d}_E$ are the molecular weights and densities of structure and reserve, respectively (Table 3).

### Estimation procedure

State variables in DEB models represent an aggregation of complex physiological functions, and therefore, model parameters cannot be associated directly with empirical data (Nisbet *et al.,* 2012). Auxiliary theory links the abstract DEB state variables to quantities that can be measured directly such as weight, length, feeding, respiration, egg production, etc. (Kooijman *et al.,* 2008; Lika *et al.,* 2011a). We used different types of empirical data (see Table 4 and Figure 2) to estimate the DEB parameters using the “add my pet” procedure (Marques *et al.,* 2018) and the covariation method (Lika *et al.,* 2011a; Marques *et al.,* 2019) implemented in MatLab (The Math Works Inc., 2020) with the software package DEBtool (available at https://www.bio.vu.nl/thb/deb/deblab/). Briefly, parameter estimates are derived through simultaneously minimizing the weighted sum of squared deviations between provided data and model estimates. Model goodness of fit was evaluated with the mean relative error (MRE) and symmetric mean squared error (SMSE) (Marques *et al.,* 2019). Lower MRE and SMSE indicate better model predictions.

### Data for model parameterization

Data used for parameter estimation were obtained from published literature from laboratory studies and surveys of wild populations (Table 4). Because there are many lake trout ecomorphs with very different life histories, only data collected specifically from the siscowet ecomorph were included for parameter estimation. Age at puberty (average age at reproductive maturity), life span (oldest recorded age), total length at puberty (total length at reproductive maturity), wet weight at puberty (wet weight at reproductive maturity), ultimate length (longest recorded length), length–number of eggs (number of eggs produced at a given length), wet weight–number of eggs (number of eggs produced at a given weight), time–length (length at age), time–wet weight (weight at age) and length–wet weight were obtained from observations of wild siscowet lake trout surveyed in Lake Superior (Miller and Schram, 2000; Goetz *et al.,* 2011, 2017; Sitar *et al.,* 2014; Hansen *et al.,* 2016; Froese and Pauly, 2021). Additional information, such as individual egg weights, were obtained from laboratory rearing studies (Smith *et al.,* 2016). Data for estradiol concentration, egg mass wet weight, muscle lipid concentration (% of total muscle mass), length at birth (length at exogenous feeding), age at birth (days from fertilization to exogenous feeding) and length–weight over the course of a single year for parasitized and unparasitized individuals were provided from a laboratory study (Firkus *et al.,* 2022).

Physical length, ${L}_w$, is linked to the structural volumetric length, $L={\left(\frac{M_V}{\left[{M}_V\right]}\right)}^{1/3}$, with the shape factor, ${\delta}_M$, which differs depending on type of measurement (standard/total): ${L}_w=L/{\delta}_M$. Mass quantified as total wet weight, ${W}_w$, has contributions from structural mass, reserve mass and ripe (${M}_{OV})$ and unripe (${M}_R)$reproductive mass: ${W}_w=\frac{w_V{M}_V}{d_V}+\frac{w_E\left({M}_E+{M}_R+{M}_{OV}\right)}{d_E}$, conversion parameters are given in Tables 2 and 3.

### Validation

After model parameterization, the resulting DEB model was validated using data collected from wild siscowet lake trout sampled near the Keweenaw Peninsula in Lake Superior (Goetz *et al.,* 2011). The validation data set included estradiol concentration, total length, total weight and gonadal weight from a population sampled monthly for 6 months leading to spawning. The data show considerable variability in length and plasma estradiol concentration, which are skewed to the right, and are therefore suited for a lognormal distribution. Using the estimated parameter set from the base DEB model (Tables 2 and 3), 200 Monte Carlo simulations were performed to introduce inter-individual variability. In each simulation three parameters were allowed to randomly vary, namely the initial fish length at the beginning of the simulation, the maximum surface-area specific assimilation rate $\left\{{\dot{p}}_{Am}\right\}$ and the conductance rate $\dot{v}$, to account respectively for the different individual sizes at the start of the experiment, assimilation (implicitly feeding) performance and lipid content. In each simulation, $\left\{{\dot{p}}_{Am}\right\}$ and $\dot{v}$ were assigned numbers randomly chosen from normal distributions with mean defined from estimated values of $\left\{{\dot{p}}_{Am}\right\}$ and $\dot{v}$ (for adults) and a 20% coefficient of variation (i.e. standard deviation equals 0.2 times the mean). As a result of the varied initial conditions, simulated individuals differed in growth and reproduction patterns. The initial length was drawn from a lognormal distribution with parameters $\mu =4.09$ and $\sigma =0.13,$ (i.e. mean 60.2 mm and standard deviation 7.81 mm). The parameters $\mu$ and $\sigma$ were obtained by fitting the lognormal distribution to the first length measurements from the validation data set. As in the DEB estimation procedure, estradiol concentration was used as forcing variable in the egg module for each simulation. At each sampling time for the 6 months leading to spawning, the lognormal distribution was fitted to the estradiol data. These distributions were then used to obtain random sets of estradiol concentration values used in the validation.

**Table 2:** Primary abj DEB parameters estimated for siscowet lake trout at a reference temperature of ${T}_{ref}=20{}^{\circ}\mathrm{C}$. Any rate parameter, $k$, can be converted to its value at any given temperature $T$ by multiplying its value with the correction factor $TC=\mathit{\exp}\left(\frac{T_A}{T_{ref}}-\frac{T_A}{T}\right)$

Symbol	Value	Unit	Interpretation
$\left\{{\dot{p}}_{Am}\right\}$	550.41	J cm^−2^ d^−1^	Maximum surface-area specific assimilation rate
$\left\{\dot{v}\right\}$	0.01644	cm d^−1^	Energy conductance
$\kappa$	0.6028	–	Allocation fraction to soma
κ_R_	0.95	–	Reproduction efficiency
$\left[{\dot{p}}_M\right]$	31.75	J cm^−3^ d^−1^	Volume-specific somatic maintenance rate
[E_G_]	5217	J cm^−3^	Specific costs for structure
${\dot{k}}_j$	0.002	d^−1^	Maturity Maintenance rate coefficient
E^b^_H_	22.28	J	Maturity threshold at birth
E^j^_H_	45.74	J	Maturity threshold at metamorphosis
E^p^_H_	4.52 10^5^	J	Maturity threshold at puberty
${\dot{h}}_a$	4.253 10^−8^	d^−2^	Weibull aging acceleration
T_A_	8000	K	Arrhenius temperature
T_Ref_	293.1	K	Reference temperature
δ_M_	0.1116	–	Shape coefficient for total length
δ_Me_	0.066	–	Shape coefficient embryo
δ_Ms_	0.04235	–	Shape coefficient for standard length
${b}_H$	1 10^8^	d^−1^	Rate of reproductive reserve ripeness
${f}_F$	0.4734	—	Scaled functional response for GSI data
${f}_{LW}$	0.7114	—	Scaled functional response for length–weight data
${f}_{LWN}$	0.8275	—	Scaled functional response for length/weight–number of eggs data
${f}_{tL}$	0.7173	—	Scaled functional response for length data
${f}_{tWw}$	1.157	—	Scaled functional response for wet weight data
${s}_m$	0.75	—	Stress factor on maintenance from parasitism

**Table 3:** Compound parameters, molecular weights, chemical potentials and densities.

Compound parameters	Value	Units	Description
$\left\{{\dot{J}}_{EAm}\right\}=\left\{{\dot{p}}_{Am}\right\}/{\mu}_E$		mol cm^−2^ d^−1^	max specific assimilation rate
$\left[{M}_V\right]={d}_V/{w}_V$		mol cm^−3^	specific structural mass
${\mathrm{m}}_{\mathrm{Em}}=\frac{\left\{{\dot{J}}_{EAm}\right\}}{\left[{M}_V\right]\dot{v}}$		mol mol^−1^	max reserve density
${\dot{j}}_{EM}=\frac{\left[{\dot{p}}_M\right]{\mathrm{y}}_{\mathrm{EV}}}{\left[{E}_G\right]}$		mol mol^−1^ d^−1^	mass-spec somatic maintenance costs
${\mathrm{y}}_{\mathrm{EV}}=\frac{\left[{E}_G\right]}{\mu_E\left[{M}_V\right]}$		mol mol^−1^	coupler of reserve invested and structure produced
${\kappa}_G$		-	Growth efficiency
Molecular weights, chemical potentials and densities			
${w}_E$	23.9	g/mol	Molecular weight of dry reserve
${w}_V$	23.9	g/mol	Molecular weight of structure
${\mu}_E$	550	KJ/mol	Chemical potential of the reserve
${d}_E$	0.2	g/cm^3^	Specific density of dry reserve
${d}_V$	0.2	g/cm^3^	Specific density of structure

### Implementing effects of parasitism, muscle lipid concentration and estradiol concentration

To assess the influence of sea lamprey parasitism on siscowet lake trout reproduction and growth, we made modifications to the parameterized base DEB model that reflect the energetic consequences of parasitism. In the context of DEB, any stressor that alters physiological processes must be reflected by a change in one or more model parameters (Jager, 2019). Therefore, we must identify the physiological mode of action (pMoA) and specific DEB parameter(s) through which sea lamprey parasitism influences life history (Ashauer and Jager, 2018). Once the appropriate DEB parameter(s) is identified, a relationship between the stressor and model parameter, termed damage, must be developed. The changes to a particular DEB parameter cannot be experimentally derived and, therefore, must be developed based on best judgement and an approximation of empirically observed changes to length, weight and ovarian mass (Firkus *et al.,* 2022). In toxicology applications, the relationship between the damage function and the change in the DEB parameter is typically expressed as ‘linear-with-threshold’ model that approximates a dose–response curve (Jager, 2019). To simplify our approach, we treat sea lamprey parasitism as a binary stressor (under the assumption that we are capturing instances of severe parasitism events that lead to reproductive disruption), and our relationships between parasitism and the target DEB parameters are therefore also binary. To implement the influence of individual variation in muscle lipid concentration, a non-linear relationship between muscle lipid and associated DEB parameters was developed where the DEB parameters are altered more dramatically as the muscle lipid deviates further from empirically derived average muscle lipid for siscowet lake trout. A detailed description of the rationale and process for implementing parasitism stress, muscle lipid variation and estradiol variation is outlined below. After identifying the different pMoAs, our goal was to explore a variety of scenarios by varying muscle lipid concentration and parasitism status. Simulations were run for a period of 365 days leading to spawning, and only for female lake trout as female fecundity is more relevant for population assessments and there is relatively little information available in the literature for siscowet lake trout milt concentrations. For each simulated individual we started the simulation year at a length of 70 cm to approximate the length of reproductively mature individuals from the laboratory study that evaluated the influence of sea lamprey parasitism on lake trout reproduction (Firkus *et al.,* 2022).

### Influence of parasitism on maintenance costs

Empirical evidence suggests siscowet lake trout reduce reproductive output (Firkus *et al.,* 2022) and plasma sex steroid concentrations (Smith *et al.,* 2016; Firkus *et al.,* 2022) following sea lamprey parasitism, often leading to skipped spawning (Goetz *et al.,* 2014, 2017; Firkus *et al.,* 2022). Thus, parasitism should alter DEB parameters in a way that leads to a marked reduction of reproductive investment. Many DEB parameters can influence reproduction, but not all are likely candidates given what we know about parasitism. Although we have observed high rates of skipped spawning in parasitized siscowets, we know that some “normal” reproductive development occurs prior to spawning, but at some point, oocytes cease further development and are resorbed (Goetz *et al.,* 2011; Sitar *et al.,* 2014); therefore, the pMoA selected should allow for these observed changes. One likely pMoA is a parasitism-driven increase in somatic maintenance costs. Energy invested to soma (κ fraction) must first pay somatic maintenance costs, but parasitism is likely to increase these costs considerably. Because sea lamprey parasitism creates an open wound in the lake trout, the costs for maintaining osmotic concentration gradients, repairing tissue, replacing lost blood cells and turning over necrotic tissue will be considerably increased (Kooijman, 2010). Thus, an increase in volume-specific somatic maintenance (${\dot{p}}_M$) is a likely pMoA.

In addition to increasing somatic maintenance, an increase in maturity maintenance is also a likely result of parasitism. Energy allocated to reproduction must pay maturity maintenance costs prior to investment in reproductive processes. Maturity maintenance encompasses the costs associated with maintaining the cumulative amount of energy that has been allocated to reach each stage of development leading to reproductive maturity. After reaching reproductive maturity, additional energy is then allocated towards reproduction. Maturity maintenance costs are proportional to the total energy invested towards reproductive maturation. Because parasitism results in an increased immune response and greater regulatory and protection costs (Goetz *et al.,* 2016), we expect maturity maintenance to increase in parasitized siscowets. Thus, an increase in maturity maintenance rate coefficient (${\dot{k}}_J$) is also a likely pMoA.

It is generally good practice to alter maturity maintenance to the same degree as somatic maintenance in the presence of stress (Jager, 2019). Therefore, for both maturity and somatic maintenance, we included a stress factor ${s}_m$ that increases both maintenance terms as follows: $\left(1+{s}_m\right){\dot{p}}_M$ and $\left(1+{s}_m\right){\dot{k}}_J$. This stress factor is zero for unparasitized cases and 0.75 for parasitized cases (Table 2). The stress factor was based on a best judgement estimate and serves as a proof of concept. This value could be altered to represent different severities of parasitism.

### Influence of parasitism on plasma estradiol concentration

In addition to muscle lipid concentrations, reproductive hormone dynamics also play a critical role in reproduction. Plasma estradiol concentration is an important predictor of the likelihood of skipping spawning for siscowet lake trout (Firkus *et al.,* 2022). To account for the importance of estradiol, we implemented an egg module in the DEB model that allows reproductive hormone dynamics to dictate the conversion of energy from the reproduction buffer into eggs. Two estradiol profiles were provided to the egg module. For parasitized fish, we provided the model with an estradiol profile that approximates plasma estradiol concentrations of parasitized lake trout observed to skip spawning in laboratory experiments (Foster *et al.,* 1993; Firkus *et al.,* 2022). In unparasitized fish, the model was provided with an estradiol profile that approximates plasma estradiol concentrations of unparasitized spawning lake trout (Foster *et al.,* 1993; Firkus *et al.,* 2022) (Figure 4A). It is likely that when lake trout skip spawning following parasitism, plasma estradiol plays a role other than gonadal development. We generally think of estradiol in terms of its role in modulating hepatic production and gonadal uptake of Vtg (Tyler and Sumpter, 1996), but it also plays a key role in the immune systems of fish (Cabas *et al.,* 2018). When estradiol is used for immune-related functions, more estradiol is required to produce the same ovarian mass as an individual not facing an immune challenge. We account for the likely reduction in estradiol availability due to an increased immune response by assuming a reduced fraction of estradiol is available for egg development following parasitism, i.e. by reducing the rate of reproductive reserve ripeness $ {b}_H $.

### Influence of muscle lipid concentration on energy mobilization and reproductive efficiency

Lipid storage also plays a key role in reproduction for siscowet lake trout. Surveys of wild lake trout found that siscowet lake trout that skipped spawning had significantly lower energy reserves than those that did not skip (Sitar *et al.,* 2014). In laboratory settings, muscle lipid concentration prior to parasitism was a significant predictor of ovarian mass and the likelihood of skipping spawning (Firkus *et al.,* 2022). Therefore, accounting for individual variation and other factors that influence muscle lipid is important for accurately modeling the influence of parasitism. Approaches have been developed to account for differences in lipid storage in a DEB context, but they require the use of simplified models that do not include all of the components of a full DEB model (Martin *et al.,* 2017).

Lipid storage has no direct analogue in the DEB framework, but is most analogous to the reserve compartment, which primarily consists of polymers and lipids (Kooijman, 2010). The energy conductance parameter $\dot{v}$ controls the rate of energy mobilization from the reserve. Increasing $\dot{v}$ increases the rate at which reserves are depleted and mobilized for use. We would expect that siscowet lake trout with low muscle lipid storage would mobilize energy from the reserve at a much lower rate to allow lipid to accumulate. Reduced energy mobilization seems especially likely given the functional role of lipid for maintaining neutral buoyancy at the depths siscowet lake trout inhabit (Henderson and Anderson, 2002; Goetz *et al.,* 2014). We implemented changes to the energy conductance parameter so that it is relative to the average muscle lipid concentration in laboratory-raised lake trout that did not skip spawning (Firkus *et al.,* 2022). As muscle lipid concentration strays from the average (55.58%), energy conductance increases or decreases following the function $\dot{v}=\dot{v}\ {\left(\frac{lipid}{55.58}\right)}^{0.35}$ where “lipid” is the muscle lipid concentration of an individual lake trout (Table 2).

Muscle lipid concentration also likely influences reproductive efficiency, represented in the DEB model as ${\kappa}_R$. Empirically, low muscle lipid concentration has been associated with an increased likelihood of skipped spawning for siscowet lake trout (Sitar *et al.,* 2014; Firkus *et al.,* 2022). Because lipid reserves are drawn upon to create reproductive mass, low muscle lipid concentrations could mean there are insufficient resources such as critical amino and fatty acids available for gamete production, and producing gametes in the absence of adequate lipid reserves results in reduced reproductive efficiency. Muscle lipid concentration influencing oocyte maturation and quality is well supported in the literature for a variety of species (Craig *et al.,* 2000; Rodríguez *et al.,* 2004; Ghaedi *et al.,* 2016). To account for the influence of low muscle lipid concentration on reproductive efficiency, the unstressed ${\kappa}_R$value from the base model is modified for lake trout with muscle lipid concentrations below average for siscowet lake charr (55.58%) using the equation: ${\kappa}_R={\kappa}_R{\left(\frac{lipid}{55.58}\right)}^2$where *lipid* is the percent muscle lipid concentration of an individual and 55.58% is the average percent muscle lipid concentration of siscowet lake trout used in laboratory sea lamprey parasitism trials (Firkus *et al.,* 2022). When muscle lipid concentrations are equal or greater than average, ${\kappa}_R$ remains unchanged. With this function, the further muscle lipid concentration deviates below average, the lower ${\kappa}_R$ is, but as muscle lipid increases above average, ${\kappa}_R$ does not increase as reproduction efficiency is rarely greater than 0.95 in DEB applications (Lika *et al.,* 2011b).

## Results

### DEB model parameters

**Figure 3:**
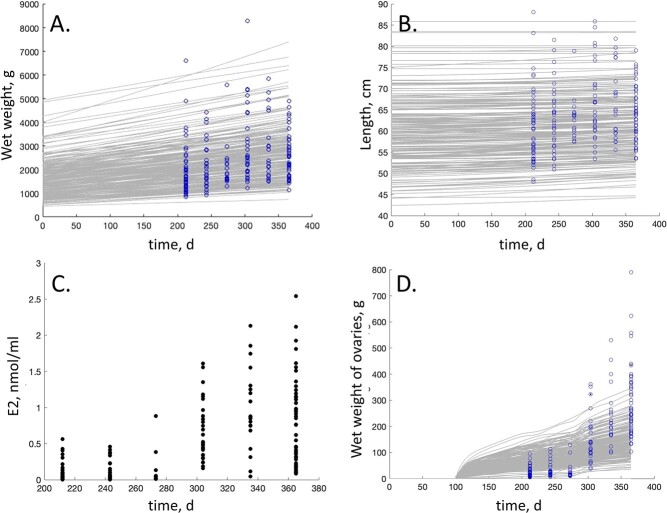
Body wet weight (A), length (B), estradiol concentration (C) and wet weight of ovaries (D) as functions of time: comparison of model predictions (solid grey lines, 200 Monte Carlo simulations) to data (blue circles) from wild siscowet lake trout (Goetz *et al.,* 2021). Estradiol data for the 6 months leading to spawning (black points).

The parameter estimates of the base siscowet lake trout DEB model are given in Table 2. Predictions from the parameterized DEB model matched the provided data well and resulted in an acceptable overall goodness of fit as measured by the MRE (0.087) and the SMSE (0.106) (Table 4). Estimates for length–time, length–weight and weight–time were all reasonable with relatively low relative error (RE < 0.15; Figure 2C, D, E). Fecundity at length was slightly overestimated (RE = 0.274) and fecundity at weight was slightly underestimated (RE = 0.237), but still followed observed trends (Figure 2A, B). The model also provided reasonable estimates of egg wet weight for siscowet lake trout (Figure 2F). Validation of the model (Figure 3) was performed by comparing the model predictions (body wet weight, length and wet weight of ovaries as functions of time) to data collected from wild siscowet lake trout sampled near the Keweenaw Peninsula in Lake Superior (Goetz *et al.,* 2011) and simulated estradiol concentrations (Figure 3C). The 200 Monte Carlo simulations captured the variation in total body wet weight and length well (Figure 3A, B), but slightly under-predicted ovarian mass in the last two months prior to spawning (Figure 3D).

**Table 4:** Comparisons of model predictions with observed life history data provided to the model and relative errors (mean of relative differences between model predictions and data used in calibration).

Data type	Observed data	Predicted estimates	Relative error	Data symbol	Units	Observed data source
age at birth (7 °C)	127	128	0.008	ab	d	(Firkus *et al.,* 2022)
age at puberty (5 °C)	4161	3804	0.086	ap	d	(Sitar *et al.,* 2014)
life span	18 250	18 270	0.001	am	d	(Froese and Pauly, 2021)
length at birth	2.775	2.771	0.001	Lb	cm	(Firkus *et al.,* 2022)
total length at puberty	44.3	37.8	0.147	Lp	cm	(Sitar *et al.,* 2014)
ultimate standard length	150	134	0.106	Li	cm	(Froese and Pauly, 2021)
egg wet weight	0.065	0.064	0.006	Ww0	g	(Smith *et al.,* 2016)
wet weight at puberty	680	732	0.077	Wwp	g	(Sitar *et al.,* 2014)
ultimate wet weight	32 700	32 700	<0.001	Wwi	g	(Froese and Pauly, 2021)
end of reproduction cycle ovarian mass	239	221.7	0.072	tMov	G	(Firkus *et al.,* 2022)

### Parasitism and muscle lipid concentration

We explored the combined influence of muscle lipid concentration and parasitism on reproduction and growth outcomes by introducing modifications to the base DEB model. Empirical evidence suggests that siscowet lake trout can overcome adverse effects on reproduction following sea lamprey parasitism if muscle lipid concentrations are sufficiently high. Additionally, unparasitized lake trout with low muscle lipid concentrations skip spawning more frequently than unparasitized lake trout (Firkus *et al.,* 2022). Therefore, our model should adequately reflect these empirical observations. In our model, we altered parasitism status and provided three different muscle lipid concentrations representing natural variation of muscle lipid in siscowet lake trout. The average muscle lipid from siscowet lake trout in laboratory studies (55.58%) (Firkus *et al.,* 2022) was used, as well as 65% and 45% representing approximate high and low bounds of natural variation (Sitar *et al.,* 2020).

**Figure 4:**
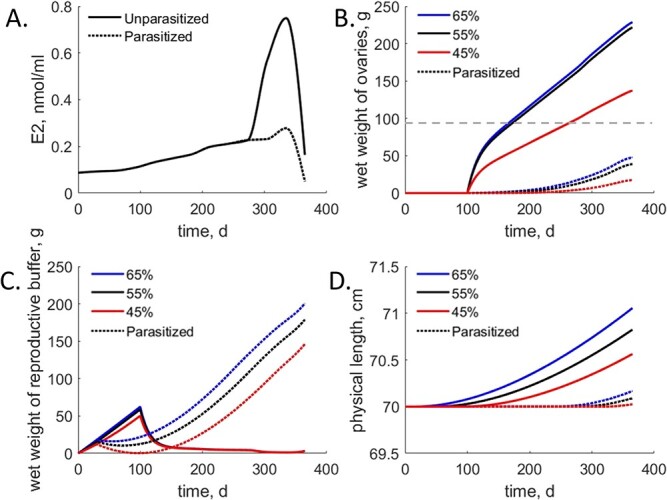
Simulation of estradiol concentrations (A), wet weight of ovaries (B), wet weight of the reproductive buffer (unripe (C) and structural length (D) under different parasitism and muscle lipid concentration scenarios. Time on the x-axis indicates days beginning 365 days prior to spawning. Muscle lipid concentrations are indicated by line color (blue, 65%; black, 55% and red, 45%) and scenarios with parasitism are indicated by dotted lines. Estradiol outcomes (A) were identical for all lipid scenarios and only differed with parasitism. The grey dashed line (B) indicates the ovarian mass threshold for skipped spawning.

For unparasitized siscowet lake trout, varying lipid altered ovarian mass matched our expectations from the empirical evidence. The muscle lipid concentration for the average siscowet lake trout in our data (55.58%) resulted in an ovarian weight of 222 g at spawning (day 365). A 10% reduction in muscle lipid resulted in a reduction of ovarian mass to 137 g, while a 10% increase in muscle lipid increased ovarian mass to 229 g (Figure 4B). Adding the influence of parasitism strongly reduced ovarian mass regardless of muscle lipid concentration, but higher muscle lipid concentration was slightly able to mitigate this reduction. Under average muscle lipid concentrations (55.58%) and parasitism, ovarian weight was 39 g at the time spawning would normally occur. A 10% reduction in muscle lipid resulted in an ovarian mass of 18 g, while a 10% increase in muscle lipid increased ovarian mass to 48 g (Figure 4B).

Differences in ovarian mass driven by parasitism and muscle lipid are observable in the wet weight of the reproductive buffer (Figure 4C). In scenarios without parasitism, the reproductive buffer builds until day 100 after which it begins to be converted into ovarian mass (Figure 4B). In scenarios with parasitism, the reproductive buffer builds more slowly initially due to higher maintenance costs. After 100 days the reproductive buffer is rapidly converted to ovarian mass in unparasitized scenarios, but in parasitized scenarios, slower conversion rates (${b}_H$) from reproductive buffer to ovarian mass (Figure 4C) result in a much smaller ovarian mass at day 365. Differences in reproductive buffer accumulation due to muscle lipid concentration are largely driven by reduced reproductive efficiency (${\kappa}_R$) and less energy being mobilized from the reserve ($\dot{v}$).

Somatic growth (increase in structural mass/physical length) was also influenced by both parasitism and muscle lipid concentration in our tested scenarios, albeit subtly. Growth was slightly lower in parasitized scenarios than in unparasitized scenarios with the same muscle lipid concentration (Figure 4D). At a 55.58% muscle lipid concentration, the scenario without parasitism resulted in growth of 8.2 mm by the end of the year, while the parasitism scenario with the same muscle lipid concentration resulted in an end-of-year increase of only 0.9 mm (Figure 4C). In the highest muscle lipid concentration scenarios (65%) lake trout grew 10.6 mm over the course of the year without parasitism, but only grew 1.7 mm when parasitized. For the lowest muscle lipid concentration scenarios (45%), lake trout grew 5.6 mm without parasitism and 0.3 mm with parasitism (Figure 4D).

## Discussion

Parasitism is a complex stressor for host species and influences multiple physiological processes simultaneously. Capturing the full extent of these effects, and their implications for the whole organism, is challenging using empirical measurements alone. DEB theory allows us to cumulatively incorporate empirical measurements of the effects of parasitism into one coherent framework that allows the consequences for many different processes to be evaluated simultaneously. In this study, we developed and parameterized a DEB model that captures the energy dynamics of siscowet lake trout. The model reproduced key life history features specific to the siscowet lake trout ecomorph and produced model estimates that adequately matched field and laboratory collected data. We also developed modifications to key DEB parameters to capture the effects of sea lamprey parasitism on reproduction and growth and account for the influence of individual variation in muscle lipid concentration and estradiol profiles observed in laboratory studies. Using these modifications, we explored several scenarios and evaluated their influence on ovarian mass and growth. We found that implementing stress from sea lamprey parasitism via increases to somatic and maturity maintenance and a reduction to estradiol concentration in our model resulted in a good approximation of observed empirical results for reproduction and growth. Altering energy conductance and reproductive efficiency with muscle lipid concentrations also represented the natural variation observed in siscowet lake trout populations well and provided insight into the modulating role muscle lipid concentrations can have in the response to sea lamprey parasitism. These findings point to the plausible physiological mechanisms at play during sea lamprey parasitism and can guide future empirical studies. Because our model can estimate reproduction and growth outcomes with and without sea lamprey parasitism and account for natural variation in lipid levels, it can help inform existing models that attempt to estimate lake trout populations under various sea lamprey control scenarios. Additionally, this work provides the foundation for future DEB models that wish to assess the effects of parasitism on other species.

### Influence of parasitism and individual variation

Studies of wild and laboratory-raised siscowet lake trout indicate unparasitized individuals skip spawning at some baseline rate as a part of their life history, and that skipping is at least partially dependent on muscle lipid concentration (Goetz *et al.,* 2011; Sitar *et al.,* 2014; Firkus *et al.,* 2022). Therefore, we would expect low lipid levels to result in lower-than-typical ovarian weight in our modeled scenarios. Under the scenarios we tested, muscle lipid concentration had a heavy influence on reproduction regardless of parasitism status. At the lowest lipid simulation (45%) without parasitism, ovarian mass reached 137 g just prior to spawning (Figure 4B). The threshold for skipping spawning is a gonadosomatic index below 3.0 (Goetz *et al.,* 2011). In our simulations this would mean any lake trout with ovarian mass lower than 96 g would be deemed a skipped spawner. Despite the reduced ovarian mass in the lowest muscle lipid scenario without parasitism, ovarian mass remained above this threshold. For ovarian mass to be below the skipped spawning threshold in an unparasitized individual, muscle lipid concentration would have to be 38% or lower.

As expected, parasitism reduced ovarian mass at all muscle lipid concentrations in our modeled scenarios (Figure 4B). Even at high muscle lipid concentrations (65%), ovarian mass after parasitism reached only 48 g, remaining well below the threshold for skipping spawning. This outcome suggests that even if an individual lake trout has exceptionally high muscle lipid concentration, it cannot overcome the energetic consequences of sea lamprey parasitism for reproduction. Average siscowet lake trout muscle lipid concentrations range from 29 to 64% in the wild depending on size and the depth inhabited by the individual (Sitar *et al.,* 2020), therefore lake trout with muscle lipid concentrations sufficiently high to mitigate the effects of sea lamprey parasitism would be rare. This outcome is consistent with laboratory studies where high muscle lipid concentrations were largely insufficient to overcome the adverse effects of sea lamprey parasitism. (Firkus *et al.,* 2022).

The alterations to DEB parameters we implemented are not necessarily an accurate representation of how sea lamprey parasitism influences the energy budget of a siscowet lake trout. Because the metabolic parameters in DEB models are abstract and include many processes that cannot be directly measured, the process for implementing stress is inherently arbitrary (Jager, 2019). Regardless, the alterations we implemented do a reasonable job of describing the effects on growth, reproduction and energy storage observed from the empirical data, and at the very least serve as plausible hypotheses for future experimental work. Applying the alterations to DEB parameters that we developed also allows us to examine the consequences of parasitism on reproduction and growth under a variety of scenarios.

Other DEB models have similarly captured the influence of parasitism on host reproduction. Hall *et al*. (2007) modeled two putative parasitism strategies and the consequences for host reproduction and growth in a generalized DEB model; one strategy where the parasite affects the host simply by draining energy resources indiscriminately, and one strategy where the parasite actively influences host energy allocation away from reproduction to provide more available energy to the parasite (influencing the κ parameter). In our model, sea lamprey parasitism acts similar to the former strategy by reducing energy available to the host through the increase of maintenance costs and reducing the efficiency of various processes such as conversion of estradiol into ovarian mass. Although sea lamprey do manipulate some physiological processes in hosts, including immune function and the clotting response (Goetz *et al.,* 2016; Bullingham *et al.,* 2021), our models suggests that sea lamprey do not actively induce energy reallocation away from reproduction in an attempt to make more energy available for consumption. Our attempts to modify energy allocation with the κ parameter resulted in more dramatic changes to lake trout mass than were observed empirically. If sea lamprey actively induced reallocation of energy away from reproduction, we would expect to observe increased growth in lake trout following parasitism, but changes in growth were not observed following parasitism for siscowet lake trout in laboratory studies (Smith *et al.,* 2016; Firkus *et al.,* 2022) suggesting that sea lamprey parasitism does not influence the allocation fraction to soma parameter κ for the siscowets.

Other approaches using DEB models to represent parasite–host dynamics have highlighted the importance of factors other than parasitism for understanding the full scope of parasitism-driven changes to reproduction. For example, variation in host food consumption can drastically change parasite virulence, host survival and reproduction (Hall *et al.,* 2009). Our model similarly highlights how muscle lipid concentration interacts with parasitism to influence reproduction. Due to the critical role muscle lipid plays in siscowet lake trout reproduction (Sitar *et al.,* 2014), including the influence of muscle lipid as a pMoA on reserve mobilization and reproduction efficiency in our DEB model allows for a more complete picture of how parasitism influences reproduction.

### Model limitations

It is important to highlight the limitations of this model and resulting simulations. First, the alterations to the DEB model implemented to represent parasitism are not directly measured. Because each DEB model parameter represents an abstracted process within the organism, changes to observed empirical endpoints often involve many DEB parameters. Thus, we were required to rely on our best judgement and implement changes to DEB parameters that matched our knowledge of the physiological modes of action caused by parasitism and that resulted in changes to endpoints we were able to empirically observe. The changes we implemented to DEB parameters are therefore presumptive and other processes that we did not consider could be important. For example, sea lamprey parasitism could potentially influence host feeding behavior, but we did not alter lake trout food intake in our model due to a lack of empirical evidence. If food intake is substantially reduced, it could further influence predicted reproductive and growth outcomes. Our model therefore only serves as a reasonable hypothesis for how parasitism, muscle lipid and estradiol concentration influence lake trout energy budgets. Likewise, our simulation results reflect the decisions we made when developing the relationships between parasitism, muscle lipid, estradiol and respective DEB parameters. Despite these limitations, our model and simulation results provide testable hypotheses that can drive empirical research going forward. For example, future work looking to identify the physiological mechanisms leading to skipped spawning in lake trout should consider mechanisms related to energy mobilization and the efficiency of processes related to egg maturation as our model hypothesizes these factors to be critical components of reduced ovarian mass. Our model also hypothesizes that sea lamprey parasitism influences hosts by increasing energetic costs associated with healing a large wound, replacing lost blood cells and mounting an immune response, but not by causing the host to reallocate energy directly away from reproduction. A study could evaluate this hypothesis by simulating the tissue damage and blood loss of sea lamprey parasitism on unwounded lake trout and observing if the changes to reproduction and growth match observations under sea lamprey parasitism.

## Conclusions

Modeling the effects of sea lamprey parasitism on lake trout in the context of DEB models is a powerful approach that accounts for the entire energy budget of the organism. Parasitism is a complex stressor that influences many different physiological functions and interacts with the life history of the host, which makes the understanding of the cumulative effects on growth and reproduction challenging. The presented DEB model for siscowet lake trout allows us to explore these cumulative effects and interactions of sea lamprey parasitism and is a step towards accounting for the sublethal effects of sea lamprey parasitism in lake trout population models.

The DEB model presented in this paper can be useful for improving existing efforts to monitor lake trout populations and direct resources for sea lamprey control in the Laurentian Great Lakes. If integrated into an individual-based model, this DEB model could allow lake trout populations to be estimated while accounting for the population-level influences of sea lamprey parasitism and individual variation both among and between lake trout ecomorphs. Additionally, simulations evaluating the effects on reproduction and growth can be developed to adjust stock-recruitment model parameters in existing models such as spawning stock biomass, or spawners per recruit. Accounting for changes in spawning stock biomass or spawners per recruit with DEB model outputs is a promising approach for incorporating the sublethal effects of parasitism and other stressors into population models going forward. Additionally, these efforts help identify knowledge gaps in our mechanistic understanding of sea lamprey parasitism and can provide us with testable hypotheses that can inform future empirical studies.

## Funding

This work was supported by a grant awarded to CM from the Great Lakes Fishery Commission. CM was also partially supported through the Michigan State University AgBioResearch through USDA National Institute of Food and Agriculture, Hatch project 1014468. TF was additionally supported by the Howard A. Tanner Fellowship.

## Conflict of interest statement

The authors have no conflicts to declare.

## Data availability

The data underlying this article are available in the article and in its online supplementary material.

## Author contribution

TF analysed and prepared data, developed the base model and parasitism model, wrote the manuscript, prepared figures and provided editorial feedback. KL developed the base model and parasitism model, developed the egg module, wrote the manuscript, prepared figures and provided editorial feedback. ND analysed and prepared data and assisted with development of the base model. CM conceived the project concept, procured funding, assisted in writing the manuscript and provided editorial feedback. All authors approved the final manuscript.

## Supplementary Material

Web_Material_coad006
