# Supplementary material for: The consequences of sea lamprey parasitism on lake trout energy budgets
Source: Conserv Physiol. 2023 Mar 8;11(1):coad006. doi: 10.1093/conphys/coad006 (PMC9994604; doi:10.1093/conphys/coad006)
Supplement: Web_Material_coad006 [file web_material_coad006.docx]

Supplementary Data 1- Matlab code for “run” file for base model

% (Salvelinus namaycush). They use functions in DEBtool package

% (https://github.com/add-my-pet/DEBtool_M)

%

% THE CONSEQUENCES OF SEA LAMPREY PARASITISM ON LAKE TROUT ENERGY BUDGETS

% Tyler J. Firkus, Konstadia Lika, Noah Dean, Cheryl A. Murphy

close all

global pets

pets = {'Salvelinus_namaycush_sisc'};

check_my_pet(pets);

estim_options('default');

estim_options('max_step_number', 500);

estim_options('max_fun_evals',5e3);

estim_options('pars_init_method', 2);

estim_options('results_output', 2);

estim_options('method', 'no');

estim_pars;

Supplementary Data 2- Matlab code for “run” file for parasitism simulation model

%% Files used for simulating the effects of parasitism on siscowet lake charr

% (Salvelinus namaycush). They use functions in DEBtool package

% (https://github.com/add-my-pet/DEBtool_M)

%

% THE CONSEQUENCES OF SEA LAMPREY PARASITISM ON LAKE TROUT ENERGY BUDGETS

% Tyler J. Firkus, Konstadia Lika, Noah Dean, Cheryl A. Murphy

close all; clear all;

[data, auxData, metaData, txtData, weights] = mydata_Salvelinus_namaycush_sisc_simu;

[par, metaPar, txtPar] = pars_init_Salvelinus_namaycush_sisc_simu(metaData);

[prdData, time_1, time_2, time_3, time_4, time_5, time_6, OUT_mR_1, M_ovaries_aux_1, ...

OUT_mR_2, M_ovaries_aux_2, OUT_mR_3, M_ovaries_aux_3, OUT_mR_4, M_ovaries_aux_4, ...

OUT_mR_5, M_ovaries_aux_5, OUT_mR_6, M_ovaries_aux_6, info] = ...

predict_Salvelinus_namaycush_sisc_simu(par, data, auxData);

figure(1)

hold on

plot(time_1, OUT_mR_1, 'b', time_1, OUT_mR_2, 'k', time_1, OUT_mR_3, 'r', time_1, OUT_mR_4, 'b:', time_1, OUT_mR_5, 'k:', time_1, OUT_mR_6, 'r:')

xlabel('time, d')

ylabel('wet weight of reproductive buffer, g')

figure(2)

hold on

plot(time_1, M_ovaries_aux_1,'b', time_2, M_ovaries_aux_2,'k', time_3, M_ovaries_aux_3,'r', time_4, M_ovaries_aux_4, 'b:', time_5, M_ovaries_aux_5, 'k:', time_6, M_ovaries_aux_6, 'r:')

xlabel('time, d')

ylabel('wet weight of ovaries, g')

figure(3)

hold on

plot(time_1, prdData.tL_1,'b', time_2, prdData.tL_2,'k', time_3, prdData.tL_3,'r', time_4, prdData.tL_4, 'b:', time_5, prdData.tL_5, 'k:', time_6, prdData.tL_6, 'r:')

xlabel('time, d')

ylabel('physical length, cm')

t_span = linspace(0,365,500)';

signal = interp1(auxData.signal.tWw_1(:,1), auxData.signal.tWw_1(:,2),t_span,'pchi');

figure(4)

hold on

plot(t_span, 1e9*signal, auxData.signal.tWw_1(:,1), 1e9*auxData.signal.tWw_1(:,2),'ok'), hold on

xlabel('time, d')

ylabel('E2, nmol/ml')

set(gca, 'FontSize', 15, 'Box', 'on')

Supplementary Data 3- Matlab code for “mydata” file for base model

function [data, auxData, metaData, txtData, weights] = mydata_Salvelinus_namaycush_sisc

%% set metadata

metaData.phylum = 'Chordata';

metaData.class = 'Actinopterygii';

metaData.order = 'Salmoniformes';

metaData.family = 'Salmonidae';

metaData.species = 'Salvelinus_namaycush';

metaData.species_en = 'Lake trout';

metaData.T_typical = C2K(5); % K, body temp

metaData.data_0 = {'ah'; 'ab'; 'ap'; 'am'; 'Lb'; 'Lp'; 'Li'; 'Ww0'; 'Wwb'; 'Wwp'; 'Wwi'; 'Ri'};

metaData.data_1 = {'LN'; 'WwN'; 'LW'; 'tL'};

metaData.COMPLETE = 2.5; % using criteria of LikaKear2011

metaData.author = {'Bas Kooijman'};

metaData.date_subm = [2019 03 13];

metaData.email = {'bas.kooijman@vu.nl'};

metaData.address = {'VU University Amsterdam'};

metaData.author_mod_1 = {'Tyler Firkus'};

metaData.date_mod_1 = [2019 04 01];

metaData.email_mod_1 = {'firkusty@msu.edu'};

metaData.address_mod_1 = {'Michigan State University'};

metaData.curator = {'Nina Marn'};

metaData.email_cur = {'nina.marn@irb.hr'};

metaData.date_acc = [2019 06 24];

metaData.author_mod_2 = {'Tyler Firkus'; "Dina Lika"};

metaData.date_mod_2 = [2022 05 13];

metaData.email_mod_2 = {'firkusty@msu.edu'};

metaData.address_mod_2 = {'Michigan State University'};

%% set data

% zero-variate data

data.ab = 127 ; units.ab = 'd'; label.ab = 'age at birth'; bibkey.ab = 'Firkus2022';

temp.ab = C2K(7); units.temp.ab = 'K'; label.temp.ab = 'temperature';

comment.ab = 'based on swim-up time (start of feeding) of my control siscowet crosses';

data.ap = 11.4*365; units.ap = 'd'; label.ap = 'age at puberty'; bibkey.ap = 'Sitar2014';

temp.ap = C2K(5); units.temp.ap = 'K'; label.temp.ap = 'temperature';

data.am = 50*365; units.am = 'd'; label.am = 'life span'; bibkey.am = 'fishbase';

temp.am = C2K(5); units.temp.am = 'K'; label.temp.am = 'temperature';

data.Lb = 27.75e-1; units.Lb = 'cm'; label.Lb = 'total length at birth'; bibkey.Lb='Firkus2022';

temp.Lb= C2K(7); units.temp.Lb = 'K'; label.temp.Lb = 'temperature'; %Osse 1995

data.Lj = 5.5; units.Lj = 'cm'; label.Lj = 'standard length at birth'; bibkey.Lj='Firkus2022';

temp.Lj= C2K(7); units.temp.Lj = 'K'; label.temp.Lj = 'temperature';

data.Lp = 44.3; units.Lp = 'cm'; label.Lp = 'total length at puberty';bibkey.Lp = 'Sitar2014';

comment.Lp = 'estimated';

data.Li = 150; units.Li = 'cm'; label.Li = 'ultimate standard length'; bibkey.Li = 'fishbase';

data.Ww0 = 6.5e-2; units.Ww0 = 'g'; label.Ww0 = 'egg wet weight'; bibkey.Ww0 = 'Smith2016';

comment.Ww0 = 'based on egg diameter of 5 mm: pi/6*0.5^3; 5mm estimate from Smith2016';

data.Wwp = 680; units.Wwp = 'g'; label.Wwp = 'wet weight at puberty'; bibkey.Wwp = 'SitaJaso2014';

comment.Wwp = 'based on length-weight regression of L50 from model 1';

data.Wwi = 32.7e3; units.Wwi = 'g'; label.Wwi = 'ultimate wet weight'; bibkey.Wwi = 'fishbase';

%% uni-variate data

% time - length

data.tL_H = [... % time since birth (y), length (mm)

3.927392739 344.3714668

8.151815182 272.8752083

8.283828383 290.6904552

7.227722772 280.8417475

6.171617162 272.9732379

6.03960396 292.7817534

5.907590759 316.550665

5.115511551 314.6096788

9.207920792 314.4070843

10.2640264 324.2557919

11.05610561 312.335392

10.92409241 302.4409372

8.151815182 332.2811489

8.01980198 354.0698624

7.887788779 373.8783779

6.96369637 356.1023429

6.96369637 381.8449172

7.227722772 397.6734307

8.283828383 399.6013463

8.01980198 413.475803

4.059405941 411.6916642

10.79207921 359.873215

10.92409241 342.0448976

10 355.9520308

9.075907591 379.7601542

13.03630363 391.4452831

16.20462046 403.1696239

16.99669967 411.0512041

17.92079208 405.0648629

17.12871287 418.9654609

21.08910891 428.6703918

18.97689769 444.6165409

19.9009901 460.4123779

20.95709571 470.2610855

21.74917492 488.0436559

21.88118812 493.9777146

22.01320132 503.8721694

22.80528053 521.6547397

23.86138614 531.5034474

23.06930693 541.4436493

25.97359736 491.7949221

27.02970297 442.2376891

28.87788779 475.8095612

29.00990099 521.3475803

27.95379538 523.3800608

27.02970297 509.5644218

34.68646865 487.4031958

32.97029703 568.6762736

31.91419142 566.748358

29.9339934 558.9255955

28.87788779 570.8590661

27.02970297 557.0891743

25.04950495 551.2466098

23.86138614 557.2460216

25.97359736 624.4681894

27.02970297 620.4555109

27.02970297 632.336699

27.95379538 624.3701598

29.00990099 624.3178773

29.00990099 650.0604516

32.04620462 622.1873673

33.89438944 645.8582492

34.02640264 715.1586446

33.10231023 738.966768

32.04620462 782.5834069

30.1980198 689.6055942

30.1980198 675.7442081

31.12211221 665.7974708

34.95049505 606.2020063

27.29372937 662.0265987

27.16171617 679.8549162

28.08580858 691.6903572

27.95379538 703.5780806

28.74587459 691.6576806

26.10561056 707.6299709

25.04950495 703.7218573

23.99339934 689.9127537

21.08910891 715.7991047

20.1650165 709.9042578

20.0330033 698.0296049

20.95709571 658.3798974

20.0330033 648.5246544

23.06930693 634.5129562

23.72937294 630.5198837

23.86138614 646.3549325

24.91749175 648.2828481

23.86138614 658.2361206

23.99339934 612.6850309

25.04950495 600.7515603

23.72937294 592.8961213

22.93729373 610.7571153

22.01320132 594.9612783

21.08910891 600.9476195

20.1650165 593.0725746

20.95709571 624.7165311

20.0330033 620.8018822

18.97689769 616.8937686

17.78877888 611.0119923

18.05280528 587.2365454

16.86468647 587.2953632

16.07260726 628.9187335

15.94059406 621.0044767

14.09240924 623.076169

12.90429043 607.2934026

15.28052805 595.294579

10.2640264 506.4340097

9.075907591 484.7106493

7.887788779 482.789269

9.207920792 456.9813417

8.943894389 441.1528282

10 472.783714

9.471947195 407.4633206

10.92409241 385.609254

9.867986799 389.6219325

15.01650165 421.0502238

16.86468647 454.6220959

16.99669967 440.7541744

18.05280528 456.5434761

18.97689769 458.477927

18.05280528 478.3256543

18.97689769 480.2601052

19.9009901 505.9569323

21.08910891 515.7991047

20.95709571 533.6274221

23.06930693 567.1862236

24.91749175 575.0155214

25.97359736 572.9830409

27.95379538 586.7463974

27.68976898 602.6010522

28.87788779 598.5818384

26.89768977 588.7788779

12.90429043 569.6696402

12.90429043 553.8280561

13.03630363 534.0195406

11.98019802 524.1708329

11.98019802 510.3094468

11.05610561 488.5730157

12.37623762 490.4878607

11.05610561 464.8106395

10.1320132 437.1336144

13.96039604 531.9935954

14.88448845 553.7300265

14.88448845 571.5518086

19.10891089 593.124857

19.10891089 565.4020848

17.92079208 565.4609025

16.20462046 563.5656635

16.20462046 547.7240793

17.52475248 549.6389243

18.97689769 551.5472339

19.9009901 549.5212888

20.95709571 559.3699964

20.95709571 575.2115806

20.0330033 523.7721792

18.97689769 531.7452537

17.78877888 529.8238735

16.46864686 527.9090285

15.28052805 527.9678463

14.09240924 512.1850799

15.54455446 510.1329935

16.46864686 512.0674444

17.39273927 512.0216972

18.58085809 509.9826814

18.58085809 496.1212953

17.39273927 494.199915

16.46864686 492.2654642

14.88448845 494.3240859

13.30033003 494.4025096

11.32013201 401.4312322

12.90429043 413.2339967

15.94059406 472.4896252

15.80858086 442.7931902

15.80858086 454.6743783

14.88448845 436.8983433

14.88448845 450.7597294

14.88448845 472.5419077

13.03630363 470.6532039

11.98019802 466.7450904

11.32013201 450.9361827

11.18811881 433.1209359

11.98019802 425.1609319

11.05610561 419.266085

10 427.2391596

10.2640264 454.9488612

13.96039604 458.7262687

13.96039604 442.8846845

13.96039604 425.0629023

13.03630363 435.0096396

12.77227723 446.9038983

3.927392739 286.9457243];

data.tL_H(:,1) = 365 * data.tL_H(:,1); % convert yr to d

data.tL_H(:,2) = 0.1 * data.tL_H(:,2); % convert mm to cm

units.tL_H = {'d', 'cm'}; label.tL_H = {'time since birth', 'length'};

temp.tL_H = C2K(5); units.temp.tL_H = 'K'; label.temp.tL_H = 'temperature';

bibkey.tL_H = {'Hansen2016'};

%time-Wet Weight

data.tWw = [... %time since birth (days), wet weight (g)

8.267368671368168, 0.3560308247935966

8.647030847087326, 0.44122258800448844

8.83652752903758, 0.42032593429295506

8.941698187519973, 0.3887282914830532

9.152764050621625, 0.46310016571670687

9.047816329412115, 0.5370261654045949

9.40618799557098, 0.5798598467677811

9.574282699324499, 0.4954409327695508

9.47000378993364, 0.6963520030913966

9.806583337668224, 0.6015887996314095

9.975068181649291, 0.5912445101696546

9.786128842881244, 0.7179620560761553

10.312483744157186, 0.6552126450021181

10.186579919296706, 0.7502730981592816

10.102476833101727, 0.7819004659389011

10.060899031709113, 0.8876619081944321

9.934437863666428, 0.8769014691566275

10.355454903505317, 0.8140034332340029

10.650122243937966, 0.7615091367125668

11.008270972823949, 0.7620144611977677

10.903211782977996, 0.8147762824466627

10.588034213440146, 0.9730617461933466

11.177926237487647, 0.9738940453454417

11.072476907414151, 0.9525812420578594

11.03128924624908, 1.1324173088498677

10.820557789056753, 1.1215379699331924

11.03212526102239, 1.2911486471423164

11.200665839321676, 1.2913864469000575

11.369206417620962, 1.2916242466577987

11.368537605802315, 1.1646391760238384

11.663260680553183, 1.1227269687219001

11.66381802373539, 1.2285478609168665

11.601507055964687, 1.3977721135196592

11.454591393135019, 1.5033849309266012

12.064269100150854, 1.2608589029999928

12.359493783765709, 1.3141854986735222

12.066219801288579, 1.6312320256823734

12.740995191986151, 1.7485862061278024

12.657560917609818, 1.9071986445413804

13.498313107968523, 1.5380145206477076

13.496919750013006, 1.2734622901602917

13.64846136125499, 2.046162877971568

13.417108206321016, 2.1199105278411494

13.71328037334562, 2.3531326402461223

13.881876685963128, 2.3639525292233605

14.493170688207362, 2.4283070886621534

14.492000267524729, 2.2060832150527245

14.490997049796757, 2.0156056091017858

14.34419285560353, 2.142382604947721

14.239022197121136, 2.1739802477576218

14.239468071666902, 2.258636961513595

15.44143437841373, 2.4719730691774346

15.442270393187039, 2.630704407469884

15.316533771281222, 2.757511128285538

15.084901944756144, 2.7783483320576368

15.275513313070812, 2.9690934627360344

15.527766837337536, 2.8636292701776807

14.644210690585359, 3.1057688734979596

15.907930621920677, 3.044059836364041

16.076025325674202, 2.9596409223658107

16.517887000527622, 2.854444254534915

16.770809336612988, 2.8759651326105207

17.171706287574217, 2.9929328884496194

17.575389954446482, 3.639005105263549

19.747077663914634, 3.9701115429488647

];

data.tWw(:,1) = data.tWw(:,1)*365; % convert years to days

data.tWw(:,2) = data.tWw(:,2)*1000; % convert kg to g

units.tWw = {'d', 'g'}; label.tWw = {'time since birth', 'wet weight in g'};

temp.tWw = C2K(5); units.temp.tWw = 'K'; label.temp.tWw = 'temperature';

bibkey.tWw = 'Miller2000';

%length-wet weight

data.LW = [...% total length (cm), weight (kg)

37.27979274611399 0.34090909090909083

39.53367875647668 0.43181818181818166

40.233160621761655 0.43181818181818166

40.777202072538856 0.3977272727272725

41.865284974093264 0.4772727272727275

41.01036269430051 0.5340909090909092

41.398963730569946 0.5227272727272725

42.25388601036269 0.5681818181818183

42.72020725388601 0.6931818181818183

43.18652849740933 0.6931818181818183

43.96373056994818 0.4886363636363633

44.27461139896373 0.6136363636363642

45.673575129533674 0.5909090909090908

44.97409326424871 0.6022727272727275

44.74093264248704 0.7272727272727266

43.96373056994818 0.704545454545455

45.284974093264246 0.9090909090909092

45.829015544041454 0.8863636363636367

45.829015544041454 0.8068181818181825

46.917098445595855 0.6590909090909092

46.917098445595855 0.8522727272727275

46.76165803108808 0.9090909090909092

48.549222797927456 0.7613636363636367

48.082901554404145 0.9659090909090908

49.71502590673575 0.75

50.56994818652849 0.954545454545455

49.870466321243526 0.954545454545455

49.170984455958546 1.0113636363636358

49.093264248704656 1.1136363636363633

48.005181347150256 1.0340909090909092

49.870466321243526 1.1363636363636367

51.34715025906735 1.1590909090909092

52.2020725388601 1.1022727272727275

52.27979274611398 1.2386363636363642

52.04663212435233 1.3977272727272725

51.58031088082902 1.5

51.19170984455958 1.295454545454545

50.33678756476684 1.3068181818181817

49.870466321243526 1.329545454545455

53.75647668393782 1.2840909090909092

54.84455958549223 1.3181818181818183

53.75647668393782 1.6363636363636358

54.611398963730565 1.4545454545454541

54.37823834196891 1.4545454545454541

56.0880829015544 1.7386363636363633

55.854922279792746 1.9090909090909092

58.652849740932645 1.5340909090909092

58.652849740932645 1.2727272727272725

59.119170984455955 2.034090909090909

58.41968911917098 2.1136363636363633

59.818652849740936 2.3636363636363633

59.27461139896373 2.329545454545455

61.6839378238342 2

61.6839378238342 2.1931818181818183

61.76165803108808 2.420454545454546

60.82901554404145 2.159090909090909

60.98445595854922 2.2727272727272725

64.48186528497409 2.454545454545454

64.48186528497409 2.6136363636363633

64.71502590673575 2.840909090909091

63.93782383419689 2.75

63.3160621761658 2.7613636363636362

63.93782383419689 2.9659090909090913

62.2279792746114 3.090909090909091

66.19170984455958 2.9431818181818183

65.64766839378238 3.022727272727273

68.13471502590673 2.8636363636363638

68.98963730569947 2.9659090909090913

67.43523316062175 2.8295454545454546

70.07772020725389 3.6363636363636362

75.12953367875647 3.943181818181818];

data.LW(:, 2) = 1000*data.LW(:, 2); %convert kg to g

units.LW = {'cm', 'g'}; label.LW = {'total length', 'wet weight'};

temp.LW = C2K(5); units.temp.LW = 'K'; label.temp.LW = 'temperature'; %assuming 5C, may have to revise

bibkey.LW = 'Miller2000';

%length - number of eggs

data.LN= [... % length (mm), number of offspring (#)

535.4085603 1206.542394

558.1712062 1623.382499

568.0933852 2459.82264

573.9299611 1738.43996

582.1011673 1598.27165

602.5291829 1224.595059

596.692607 1666.907972

599.6108949 1829.472446

636.381323 2408.017374

636.9649805 2221.925618

603.6964981 3526.830151

646.3035019 3430.504027

650.3891051 3965.071034

675.4863813 2893.358067

694.7470817 2519.771966

691.8287938 2752.55633

715.7587549 3006.515247

677.2373541 3242.059542

683.07393 3567.18849

687.7431907 3869.152113

774.1245136 4815.944258

782.2957198 4257.171297

796.3035019 7930.504027

744.9416342 8074.020451];

data.LN(:,1) = 0.1 * data.LN(:,1); % convert mm to cm

units.LN = {'cm', '#'}; label.LN = {'length', 'fecundity'};

temp.LN = C2K(7); units.temp.LN = 'K'; label.temp.LN = 'temperature';

bibkey.LN = {'Goetz2011'};

comment.LN ='data extracted from figure, temperature estimated and may need to be revised';

%wet weight-number of eggs

data.WwN = [... $ wet weight (kg), number of eggs (#)

1.2949640287769792, 976.3535784917876

1.1654676258992787, 1956.9491816905029

1.4244604316546763, 1932.0129553727493

1.4820143884892083, 1883.9748917997058

1.71223021582734, 1500.5875777465662

1.6546762589928061, 1524.7212588494913

1.956834532374101, 1595.230588437611

2, 1786.2936742239617

2.0719424460431632, 1857.7201983433115

1.4964028776978413, 2481.5271288944896

1.4676258992805735, 2720.6856029120936

1.5827338129496393, 3030.983977758031

1.6258992805755401, 2672.246266731632

1.7266187050359711, 2026.4266674309947

1.8273381294964022, 2408.49551434549

1.899280575539569, 2216.9738312935297

1.6978417266187051, 2337.2982888589468

2.4460431654676267, 1808.4209922898299

2.431654676258992, 2238.757201410186

2.4748201438848927, 2429.820287196537

2.388489208633093, 2573.590529966463

2.374100719424458, 2764.88291438562

2.273381294964029, 2693.5710395826754

2.187050359712229, 2909.0544297629604

2.143884892086332, 3028.74831608816

2.057553956834532, 2957.379116627013

2.100719424460431, 2503.0238757201405

2.086330935251798, 2168.4198457966704

2.23021582733813, 2167.846599214652

2.273381294964029, 2335.0053025308807

2.7482014388489215, 2548.2530310412985

2.6330935251798557, 2716.0423055977517

2.8633093525179856, 2834.647023417121

2.7625899280575528, 2787.2395310842912

2.7050359712230208, 3098.225801828652

2.7050359712230208, 3337.2696265298473

2.6330935251798557, 3504.886927111693

2.374100719424458, 3673.2494482501643

2.215827338129495, 4104.158903952533

2.215827338129495, 4391.011493593967

3.0359712230215816, 3670.6125139728865

3.0215827338129486, 3933.618045802399

3.0359712230215816, 4076.9870159649145

3.3812949640287773, 3788.7586345266373

3.4676258992805753, 3597.1796268164762

3.3812949640287773, 2808.678953251736

3.0359712230215816, 2953.481039869297

4.172661870503598, 2207.916535297656

3.9999999999999982, 4097.05064633552

4.244604316546761, 4024.3629797357316

4.273381294964027, 4143.770242769926

4.330935251798559, 4406.489151308433

4.086330935251796, 4837.742554960014

4.647482014388489, 4261.801714007277

5.784172661870501, 4950.500157642808

5.956834532374101, 4854.194731863909

5.5107913669064725, 3397.8044655908707

3.956834532374101, 5603.198715927656

3.568345323741008, 6202.35604345209

2.374100719424458, 5346.5562211585275

4.848920863309351, 6436.29797357333

6.115107913669062, 5714.121929547995

7.007194244604316, 3941.643497950641

8.37410071942446, 4007.910802831837

7.841726618705033, 8097.68121757574];

data.WwN(:, 1) = 1000*data.WwN(:, 1);

units.WwN = {'g', '#'}; label.WwN = {'wet weight', 'fecundity'};

temp.WwN = C2K(7); units.temp.WwN = 'K'; label.temp.WwN = 'temperature';

bibkey.WwN = {'Goetz2017'};

comment.WwN ='data extracted from figure, temperature estimated and may need to be revised';

%% No parasitism, No skipped spawning CONTROL

% wet weight, length, mass of eggs, estradiol

data.tWw_1 =[... % time (d), wet weight with ovaries(kg)

1 273 304 335 365;

3.52 3.31 3.05 3.21 3.30]';

data.tWw_1(:,2) = data.tWw_1(:,2)*1000; % convert kg to g

units.tWw_1 = {'d', 'g'}; label.tWw_1 = {'time since birth', 'wet weight in g'}; bibkey.tWw_1 = 'Firkus2022';

temp.tWw_1 = C2K(8); units.temp.tWw_1 = 'K'; label.temp.tWw_1 = 'temperature';

lipid.tWw_1 = 55.58; % muscle lipid (%)

data.tL_1 =[... % time (d), wet weight with ovaries(kg)

1 273 304 335 365;

70.22 70.14 68.94 70.04 70.06]';

units.tL_1 = {'d', 'cm'}; label.tL_1 = {'time since birth', 'lengtht in cm'}; bibkey.tL_1 = 'Firkus2022';

temp.tL_1 = C2K(8); units.temp.tL_1 = 'K'; label.temp.tL_1 = 'temperature';

length0.tL_1 = 70; % initial legth (cm)

data.tMov_1 = 239;

units.tMov_1 = 'g'; label.tMov_1 = 'egg wet mass '; bibkey.tMov_1 = 'Firkus2022';

temp.tMov_1 = C2K(8); units.temp.tMov_1 = 'K'; label.temp.tMov_1 = 'temperature';

% t - estradiol data

signal.tWw_1 = [... % time (d), E2 (ng/ml)

1 31 61 92 123 152 182 213 243 273 304 335 365;

1.32 1.40 1.45 1.65 2.00 2.25 2.50 3.00 3.20 3.43 8.54 11.32 2.47]';

signal.tWw_1(:,2) = 1e-9 * signal.tWw_1(:,2)/15.1111; % E2 in C-mol/ml

%% set weights for all real data

weights = setweights(data, []);

weights.ab = 5 * weights.ab;

weights.Ww0 = 5 * weights.Ww0;

weights.Wwi = 5 * weights.Wwi;

weights.tWw = 5 * weights.tWw;

%

%% set pseudodata and respective weights

[data, units, label, weights] = addpseudodata(data, units, label, weights);

data.psd.k = 0.3; units.psd.k = '-'; label.psd.k = 'maintenance ratio';

weights.psd.k = 5*0.1;

weights.psd.v = 0 * weights.psd.v;

%% pack auxData and txtData for output

auxData.temp = temp;

auxData.signal = signal;

auxData.lipid = lipid;

auxData.length0 = length0;

txtData.units = units;

txtData.label = label;

txtData.bibkey = bibkey;

txtData.comment = comment;

%% plots

%

% set1 = {'tWw_1', 'tWw_2','tWw_3','tWw_4'}; comment1 = {'control, noPar-noSpawning, Par-noSpawning, Par-Spawning'};

% set2 = {'tL_1', 'tL_2','tL_3', 'tL_4'}; comment2 = {'control, noPar-noSpawning, Par-noSpawning, Par-Spawning'};

%

% % set2 = {'tWw'}; comment2 = {'Wet weight, Firkus'}; % <- if you want to include both of your tWw data

%

% metaData.grp.sets = {set1, set2};

% metaData.grp.comment = {comment1, comment2};

%% Discussion points

D1 = 'temperatures are guessed; 10 C is the preferred temperature';

D2 = 'Difference to version 2019/03/13 : added data on ab, tWw (Firkus) and fecundity (LN, Goetz2011) ';

metaData.discussion = struct('D1', D1, 'D2', D2);

%% References

bibkey = 'Wiki'; type = 'Misc'; bib = ...

'howpublished = {\url{https://en.wikipedia.org/wiki/Salvelinus_namaycush}}';

metaData.biblist.(bibkey) = ['''@', type, '{', bibkey, ', ' bib, '}'';'];

%

bibkey = 'Kooy2010'; type = 'Book'; bib = [ ... % used in setting of chemical parameters and pseudodata

'author = {Kooijman, S.A.L.M.}, ' ...

'year = {2010}, ' ...

'title = {Dynamic Energy Budget theory for metabolic organisation}, ' ...

'publisher = {Cambridge Univ. Press, Cambridge}, ' ...

'pages = {Table 4.2 (page 150), 8.1 (page 300)}, ' ...

'howpublished = {\url{http://www.bio.vu.nl/thb/research/bib/Kooy2010.html}}'];

metaData.biblist.(bibkey) = ['''@', type, '{', bibkey, ', ' bib, '}'';'];

%

bibkey = 'fishbase'; type = 'Misc'; bib = ...

'howpublished = {\url{https://www.fishbase.se/summary/Salvelinus-namaycush.html}}';

metaData.biblist.(bibkey) = ['''@', type, '{', bibkey, ', ' bib, '}'';'];

%

bibkey = 'SitaJaso2014'; type = 'Article'; bib = [ ...

'author = {S. Sitar and A.J. Jasonowicz and C.A. Murphy and F.W. Goetz }, ' ...

'year = {2014}, ' ...

'title = {Estimates of skipped spawning in lean and siscowet lake trout in southern lake superior: implications for stock assessment}, ' ...

'journal = {T. Am. Fish Soc.}, ' ...

'volume = {143(3)}, '...

'pages = {660-672}'];

metaData.biblist.(bibkey) = ['''@', type, '{', bibkey, ', ' bib, '}'';'];

%

bibkey = 'Hansen2016'; type = 'Article'; bib = [...

'author = {M. Hansen and N. Nate and A. Muir and C. Bronte and M. Zimmerman and C. Krueger}, ' ...

'year = {2016}, ' ...

'title = {Life history variation among four lake trout morphs at Isle Royale, Lake Superior}, ' ...

'journal = {Journal of Great Lakes Research}, ' ...

'volume = {42(2)}, '...

'pages = {421-432}'];

metaData.biblist.(bibkey) = ['''@', type, '{', bibkey, ', ' bib, '}'';'];

%

bibkey = 'Sitar2014'; type = 'Article'; bib = [...

'author = {M. Hansen and N. Nate and A. Muir and C. Bronte and M. Zimmerman and C. Krueger}, ' ...

'year = {2016}, ' ...

'title = {Life history variation among four lake trout morphs at Isle Royale, Lake Superior}, ' ...

'journal = {Journal of Great Lakes Research}, ' ...

'volume = {42(2)}, '...

'pages = {421-432}'];

metaData.biblist.(bibkey) = ['''@', type, '{', bibkey, ', ' bib, '}'';'];

%

bibkey = 'Miller2000'; type = 'Article'; bib = [...

'author = {M. Miller and S. Schram}, ' ...

'year = {2000}, ' ...

'title = {Growth and contaminant dynamics of Lake Superior lake trout}, ' ...

'journal = {Journal of Great Lakes Research}, ' ...

'volume = {26(1)}, '...

'pages = {102-111}'];

metaData.biblist.(bibkey) = ['''@', type, '{', bibkey, ', ' bib, '}'';'];

%

bibkey = 'Goetz2017'; type = 'Article'; bib = [...

'author = {F. Goetz, S. Sitar, A. Jasonowicz, M. Seider}, ' ...

'year = {2017}, ' ...

'title = {Reproduction of Lake Trout Morphotypes at Isle Royale in Northern Lake Superior}, ' ...

'journal = {Transactions of the American Fisheries Society}, ' ...

'volume = {146(2)}, '...

'pages = {268-282}'];

metaData.biblist.(bibkey) = ['''@', type, '{', bibkey, ', ' bib, '}'';'];

%

bibkey = 'Goetz2011'; type = 'Article'; bib = [...

'author = {F. Goetz, S. Sitar, D. Rosauer, P. Swanson, C. Bronte, J. Dickey, C. Simchick}, ' ...

'year = {2017}, ' ...

'title = {The Reproductive Biology of Siscowet and Lean Lake Trout in Souther Lake Superior}, ' ...

'journal = {Transactions of the American Fisheries Society}, ' ...

'volume = {140(6)}, '...

'pages = {1472-1491}'];

metaData.biblist.(bibkey) = ['''@', type, '{', bibkey, ', ' bib, '}'';'];

%

bibkey = 'Firkus2022'; type = 'Article'; bib = [...

'author = {T. Firkus, F. Goetz, G. Fischer, C. Murphy}, ' ...

'year = {2022}, ' ...

'title = {The influence of life history on the response to parasitism: differential response to non-lethal sea lamprey parasitism by two lake charr ecomorphs}, ' ...

'journal = {Integrative and Comparative Biology}, ' ...

'volume = {62(1)}, '...

'pages = {104-120}'];

metaData.biblist.(bibkey) = ['''@', type, '{', bibkey, ', ' bib, '}'';'];

%

bibkey = 'Smith2016'; type = 'Article'; bib = [...

'author = {S. Smith, S. Sitar, F. Goetz, M. Huertas, B. Armstrong, C. Murphy}, ' ...

'year = {2016}, ' ...

'title = {Differential physiological response to sea lamprey parasitism between lake trout (Salvelinus namaycush) morphotypes from Lake Superior}, ' ...

'journal = {Canadian Journal of Fisheries and Aquatic Sciences}, ' ...

'volume = {73(12)}, '...

'pages = {1815-1829}'];

metaData.biblist.(bibkey) = ['''@', type, '{', bibkey, ', ' bib, '}'';'];

Supplementary Data 4- Matlab code for “mydata” file for parasitism simulation model

function [data, auxData, metaData, txtData, weights] = mydata_Salvelinus_namaycush_sisc_simu

%% set metadata

metaData.phylum = 'Chordata';

metaData.class = 'Actinopterygii';

metaData.order = 'Salmoniformes';

metaData.family = 'Salmonidae';

metaData.species = 'Salvelinus_namaycush';

metaData.species_en = 'Lake trout';

metaData.T_typical = C2K(5); % K, body temp

metaData.data_0 = {'ah'; 'ab'; 'ap'; 'am'; 'Lb'; 'Lp'; 'Li'; 'Ww0'; 'Wwb'; 'Wwp'; 'Wwi'; 'Ri'};

metaData.data_1 = {'LN'; 'WwN'; 'LW'; 'tL'};

metaData.COMPLETE = 2.5; % using criteria of LikaKear2011

metaData.author = {'Bas Kooijman'};

metaData.date_subm = [2019 03 13];

metaData.email = {'bas.kooijman@vu.nl'};

metaData.address = {'VU University Amsterdam'};

metaData.author_mod_1 = {'Tyler Firkus'};

metaData.date_mod_1 = [2019 04 01];

metaData.email_mod_1 = {'firkusty@msu.edu'};

metaData.address_mod_1 = {'Michigan State University'};

metaData.curator = {'Nina Marn'};

metaData.email_cur = {'nina.marn@irb.hr'};

metaData.date_acc = [2019 06 24];

%% 65% lipid, no parasitism, base E2 regime

% wet weight, length, mass of eggs, estradiol

data.tWw_1 =[... % time (d), wet weight with ovaries(kg)

1 273 304 335 365;

3.2 3.2 3.2 3.2 3.2]';

data.tWw_1(:,2) = data.tWw_1(:,2)*1000; % convert kg to g

units.tWw_1 = {'d', 'g'}; label.tWw_1 = {'time since birth', 'wet weight in g'}; bibkey.tWw_1 = 'Firkus2022';

temp.tWw_1 = C2K(8); units.temp.tWw_1 = 'K'; label.temp.tWw_1 = 'temperature';

lipid.tWw_1 = 65; % muscle lipid (%)

data.tL_1 =[... % time (d), wet weight with ovaries(kg)

1 273 304 335 365;

70 70 70 70 70]';

units.tL_1 = {'d', 'cm'}; label.tL_1 = {'time since birth', 'lengtht in cm'}; bibkey.tL_1 = 'Firkus2022';

temp.tL_1 = C2K(8); units.temp.tL_1 = 'K'; label.temp.tL_1 = 'temperature';

length0.tL_1 = 70; % initial legth (cm)

% t - estradiol data

signal.tWw_1 = [... % time (d), E2 (ng/ml)

1 31 61 92 123 152 182 213 243 273 304 335 365;

1.32 1.40 1.45 1.65 2.00 2.25 2.50 3.00 3.20 3.43 8.54 11.32 2.47]';

signal.tWw_1(:,2) = 1e-9 * signal.tWw_1(:,2)/15.1111; % E2 in C-mol/ml

%% Scenario 2 - No parasitism, 55% lipid, base estradiol

data.tWw_2 =[... % time (d), wet weight with ovaries(kg)

1 273 304 335 365;

3.2 3.2 3.2 3.2 3.2]';

data.tWw_2(:,2) = data.tWw_2(:,2)*1000; % convert kg to g

units.tWw_2 = {'d', 'g'}; label.tWw_2 = {'time since birth', 'wet weight in g'}; bibkey.tWw_2 = 'Firkus2022';

temp.tWw_2 = C2K(8); units.temp.tWw_2 = 'K'; label.temp.tWw_2 = 'temperature';

lipid.tWw_2 = 55.58; % muscle lipid (%)

data.tL_2 =[... % time (d), total length(cm)

1 273 304 335 365;

70 70 70 70 70]';

units.tL_2 = {'d', 'cm'}; label.tL_2 = {'time since birth', 'lengtht in cm'}; bibkey.tL_2 = 'Firkus2022';

temp.tL_2 = C2K(8); units.temp.tL_2 = 'K'; label.temp.tL_2 = 'temperature';

length0.tL_2 = 70; % initial legth (cm)

% t - estradiol data

signal.tWw_2 = [... % time (d), E2 (ng/ml)

1 31 61 92 123 152 182 213 243 273 304 335 365;

1.32 1.40 1.45 1.65 2.00 2.25 2.50 3.00 3.20 3.43 8.54 11.32 2.47]';

signal.tWw_2(:,2) = 1e-9 * signal.tWw_2(:,2)/15.1111; % E2 in C-mol/ml

%% Scenario 3 - No parasitism, 45% lipid, base estradiol

data.tWw_3 =[... % time (d), wet weight with ovaries(kg)

1 273 304 335 365;

3.2 3.2 3.2 3.2 3.2]'; % mean 3.2425

data.tWw_3(:,2) = data.tWw_3(:,2)*1000; % convert kg to g

units.tWw_3 = {'d', 'g'}; label.tWw_3 = {'time since birth', 'wet weight in g'}; bibkey.tWw_3 = 'Firkus2022';

temp.tWw_3 = C2K(8); units.temp.tWw_3 = 'K'; label.temp.tWw_3 = 'temperature';

lipid.tWw_3 = 45; % muscle lipid (%)

data.tL_3 =[... % time (d), wet weight with ovaries(kg)

1 273 304 335 365;

70 70 70 70 70]';

units.tL_3 = {'d', 'cm'}; label.tL_3 = {'time since birth', 'lengtht in cm'}; bibkey.tL_3 = 'Firkus2022';

temp.tL_3 = C2K(8); units.temp.tL_3 = 'K'; label.temp.tL_3 = 'temperature';

% t - estradiol data

signal.tWw_3 = [... % time (d), E2 (ng/ml)

1 31 61 92 123 152 182 213 243 273 304 335 365;

1.32 1.40 1.45 1.65 2.00 2.25 2.50 3.00 3.20 3.43 8.54 11.32 2.47]';

signal.tWw_3(:,2) = 1e-9 * signal.tWw_3(:,2)/15.1111; % E2 in C-mol/ml

%% Scenario 4 - Parasitism, 65% lipid, base estradiol

data.tWw_4 =[... % time (d), wet weight with ovaries(kg)

1 273 304 335 365;

3.2 3.2 3.2 3.2 3.2]'; % mean 3.2425

data.tWw_4(:,2) = data.tWw_4(:,2)*1000; % convert kg to g

units.tWw_4 = {'d', 'g'}; label.tWw_4 = {'time since birth', 'wet weight in g'}; bibkey.tWw_4 = 'Firkus2022';

temp.tWw_4 = C2K(8); units.temp.tWw_4 = 'K'; label.temp.tWw_4 = 'temperature';

lipid.tWw_4 = 65; % muscle lipid (%)

data.tL_4 =[... % time (d), wet weight with ovaries(kg)

1 273 304 335 365;

70 70 70 70 70]';

units.tL_4 = {'d', 'cm'}; label.tL_4 = {'time since birth', 'lengtht in cm'}; bibkey.tL_4 = 'Firkus2022';

temp.tL_4 = C2K(8); units.temp.tL_4 = 'K'; label.temp.tL_4 = 'temperature';

signal.tWw_4 = [... % time (d), E2 (ng/ml)

1 31 61 92 123 152 182 213 243 273 304 335 365;

1.32 1.40 1.45 1.65 2.00 2.25 2.50 3.00 3.20 3.43 3.5 4.2 0.75]';

signal.tWw_4(:,2) = 1e-9 * signal.tWw_4(:,2)/15.1111; % E2 in C-mol/ml

%% Scenario 5 - Parasitism, 55% lipid, base estradiol

data.tWw_5 =[... % time (d), wet weight with ovaries(kg)

1 273 304 335 365;

3.2 3.2 3.2 3.2 3.2]'; % mean 3.2425

data.tWw_5(:,2) = data.tWw_5(:,2)*1000; % convert kg to g

units.tWw_5 = {'d', 'g'}; label.tWw_5 = {'time since birth', 'wet weight in g'}; bibkey.tWw_5 = 'Firkus2022';

temp.tWw_5 = C2K(8); units.temp.tWw_5 = 'K'; label.temp.tWw_5 = 'temperature';

lipid.tWw_5 = 55.58; % muscle lipid (%)

data.tL_5 =[... % time (d), wet weight with ovaries(kg)

1 273 304 335 365;

70 70 70 70 70]';

units.tL_5 = {'d', 'cm'}; label.tL_5 = {'time since birth', 'lengtht in cm'}; bibkey.tL_5 = 'Firkus2022';

temp.tL_5 = C2K(8); units.temp.tL_5 = 'K'; label.temp.tL_5 = 'temperature';

signal.tWw_5 = [... % time (d), E2 (ng/ml)

1 31 61 92 123 152 182 213 243 273 304 335 365;

1.32 1.40 1.45 1.65 2.00 2.25 2.50 3.00 3.20 3.43 3.5 4.2 0.75]';

signal.tWw_5(:,2) = 1e-9 * signal.tWw_5(:,2)/15.1111; % E2 in C-mol/ml

%% Scenario 6 - Parasitism, 45% lipid, base estradiol

data.tWw_6 =[... % time (d), wet weight with ovaries(kg)

1 273 304 335 365;

3.2 3.2 3.2 3.2 3.2]'; % mean 3.2425

data.tWw_6(:,2) = data.tWw_6(:,2)*1000; % convert kg to g

units.tWw_6 = {'d', 'g'}; label.tWw_6 = {'time since birth', 'wet weight in g'}; bibkey.tWw_6 = 'Firkus2022';

temp.tWw_6 = C2K(8); units.temp.tWw_6 = 'K'; label.temp.tWw_6 = 'temperature';

lipid.tWw_6 = 45; % muscle lipid (%)

data.tL_6 =[... % time (d), wet weight with ovaries(kg)

1 273 304 335 365;

70 70 70 70 70]';

units.tL_6 = {'d', 'cm'}; label.tL_6 = {'time since birth', 'lengtht in cm'}; bibkey.tL_6 = 'Firkus2022';

temp.tL_6 = C2K(8); units.temp.tL_6 = 'K'; label.temp.tL_6 = 'temperature';

signal.tWw_6 = [... % time (d), E2 (ng/ml)

1 31 61 92 123 152 182 213 243 273 304 335 365;

1.32 1.40 1.45 1.65 2.00 2.25 2.50 3.00 3.20 3.43 3.5 4.2 0.75]';

signal.tWw_6(:,2) = 1e-9 * signal.tWw_6(:,2)/15.1111; % E2 in C-mol/ml

%% set weights for all real data

weights = setweights(data, []);

%

%% set pseudodata and respective weights

[data, units, label, weights] = addpseudodata(data, units, label, weights);

%% pack auxData and txtData for output

auxData.temp = temp;

auxData.signal = signal;

auxData.lipid = lipid;

auxData.length0 = length0;

txtData.units = units;

txtData.label = label;

txtData.bibkey = bibkey;

%% Discussion points

D1 = 'temperatures are guessed; 10 C is the preferred temperature';

D2 = 'Difference to version 2019/03/13 : added data on ab, tWw (Firkus) and fecundity (LN, Goetz2011) ';

metaData.discussion = struct('D1', D1, 'D2', D2);

%% References

bibkey = 'Wiki'; type = 'Misc'; bib = ...

'howpublished = {\url{https://en.wikipedia.org/wiki/Salvelinus_namaycush}}';

metaData.biblist.(bibkey) = ['''@', type, '{', bibkey, ', ' bib, '}'';'];

%

bibkey = 'Kooy2010'; type = 'Book'; bib = [ ... % used in setting of chemical parameters and pseudodata

'author = {Kooijman, S.A.L.M.}, ' ...

'year = {2010}, ' ...

'title = {Dynamic Energy Budget theory for metabolic organisation}, ' ...

'publisher = {Cambridge Univ. Press, Cambridge}, ' ...

'pages = {Table 4.2 (page 150), 8.1 (page 300)}, ' ...

'howpublished = {\url{http://www.bio.vu.nl/thb/research/bib/Kooy2010.html}}'];

metaData.biblist.(bibkey) = ['''@', type, '{', bibkey, ', ' bib, '}'';'];

%

bibkey = 'fishbase'; type = 'Misc'; bib = ...

'howpublished = {\url{https://www.fishbase.se/summary/Salvelinus-namaycush.html}}';

metaData.biblist.(bibkey) = ['''@', type, '{', bibkey, ', ' bib, '}'';'];

%

bibkey = 'SitaJaso2014'; type = 'Article'; bib = [ ...

'author = {S. Sitar and A.J. Jasonowicz and C.A. Murphy and F.W. Goetz }, ' ...

'year = {2014}, ' ...

'title = {Estimates of skipped spawning in lean and siscowet lake trout in southern lake superior: implications for stock assessment}, ' ...

'journal = {T. Am. Fish Soc.}, ' ...

'volume = {143(3)}, '...

'pages = {660-672}'];

metaData.biblist.(bibkey) = ['''@', type, '{', bibkey, ', ' bib, '}'';'];

%

bibkey = 'Hansen2016'; type = 'Article'; bib = [...

'author = {M. Hansen and N. Nate and A. Muir and C. Bronte and M. Zimmerman and C. Krueger}, ' ...

'year = {2016}, ' ...

'title = {Life history variation among four lake trout morphs at Isle Royale, Lake Superior}, ' ...

'journal = {Journal of Great Lakes Research}, ' ...

'volume = {42(2)}, '...

'pages = {421-432}'];

metaData.biblist.(bibkey) = ['''@', type, '{', bibkey, ', ' bib, '}'';'];

%

bibkey = 'Sitar2014'; type = 'Article'; bib = [...

'author = {M. Hansen and N. Nate and A. Muir and C. Bronte and M. Zimmerman and C. Krueger}, ' ...

'year = {2016}, ' ...

'title = {Life history variation among four lake trout morphs at Isle Royale, Lake Superior}, ' ...

'journal = {Journal of Great Lakes Research}, ' ...

'volume = {42(2)}, '...

'pages = {421-432}'];

metaData.biblist.(bibkey) = ['''@', type, '{', bibkey, ', ' bib, '}'';'];

%

bibkey = 'Miller2000'; type = 'Article'; bib = [...

'author = {M. Miller and S. Schram}, ' ...

'year = {2000}, ' ...

'title = {Growth and contaminant dynamics of Lake Superior lake trout}, ' ...

'journal = {Journal of Great Lakes Research}, ' ...

'volume = {26(1)}, '...

'pages = {102-111}'];

metaData.biblist.(bibkey) = ['''@', type, '{', bibkey, ', ' bib, '}'';'];

%

bibkey = 'Goetz2017'; type = 'Article'; bib = [...

'author = {F. Goetz, S. Sitar, A. Jasonowicz, M. Seider}, ' ...

'year = {2017}, ' ...

'title = {Reproduction of Lake Trout Morphotypes at Isle Royale in Northern Lake Superior}, ' ...

'journal = {Transactions of the American Fisheries Society}, ' ...

'volume = {146(2)}, '...

'pages = {268-282}'];

metaData.biblist.(bibkey) = ['''@', type, '{', bibkey, ', ' bib, '}'';'];

%

bibkey = 'Goetz2011'; type = 'Article'; bib = [...

'author = {F. Goetz, S. Sitar, D. Rosauer, P. Swanson, C. Bronte, J. Dickey, C. Simchick}, ' ...

'year = {2017}, ' ...

'title = {The Reproductive Biology of Siscowet and Lean Lake Trout in Souther Lake Superior}, ' ...

'journal = {Transactions of the American Fisheries Society}, ' ...

'volume = {140(6)}, '...

'pages = {1472-1491}'];

metaData.biblist.(bibkey) = ['''@', type, '{', bibkey, ', ' bib, '}'';'];

%

bibkey = 'Firkus2022'; type = 'Article'; bib = [...

'author = {T. Firkus, F. Goetz, G. Fischer, C. Murphy}, ' ...

'year = {2022}, ' ...

'title = {The influence of life history on the response to parasitism: differential response to non-lethal sea lamprey parasitism by two lake charr ecomorphs}, ' ...

'journal = {Integrative and Comparative Biology}, ' ...

'volume = {62(1)}, '...

'pages = {104-120}'];

metaData.biblist.(bibkey) = ['''@', type, '{', bibkey, ', ' bib, '}'';'];

%

bibkey = 'Smith2016'; type = 'Article'; bib = [...

'author = {S. Smith, S. Sitar, F. Goetz, M. Huertas, B. Armstrong, C. Murphy}, ' ...

'year = {2016}, ' ...

'title = {Differential physiological response to sea lamprey parasitism between lake trout (Salvelinus namaycush) morphotypes from Lake Superior}, ' ...

'journal = {Canadian Journal of Fisheries and Aquatic Sciences}, ' ...

'volume = {73(12)}, '...

'pages = {1815-1829}'];

metaData.biblist.(bibkey) = ['''@', type, '{', bibkey, ', ' bib, '}'';'];

Supplementary Data 5- Matlab code for “pars_init” file for base model

function [par, metaPar, txtPar] = pars_init_Salvelinus_namaycush_sisc(metaData)

metaPar.model = 'abj';

%% reference parameter (not to be changed)

par.T_ref = 293.15; free.T_ref = 0; units.T_ref = 'K'; label.T_ref = 'Reference temperature';

%% core primary parameters

par.T_A = 8000; free.T_A = 0; units.T_A = 'K'; label.T_A = 'Arrhenius temp';

par.z = 11.9994; free.z = 1; units.z = '-'; label.z = 'zoom factor';

par.F_m = 6.5; free.F_m = 0; units.F_m = 'l/d.cm^2'; label.F_m = '{F_m}, max spec searching rate';

par.kap_X = 0.8; free.kap_X = 0; units.kap_X = '-'; label.kap_X = 'digestion efficiency of food to reserve';

par.kap_P = 0.1; free.kap_P = 0; units.kap_P = '-'; label.kap_P = 'faecation efficiency of food to faeces';

par.v = 0.016443; free.v = 1; units.v = 'cm/d'; label.v = 'energy conductance';

par.kap = 0.60285; free.kap = 1; units.kap = '-'; label.kap = 'allocation fraction to soma';

par.kap_R = 0.95; free.kap_R = 0; units.kap_R = '-'; label.kap_R = 'reproduction efficiency';

par.p_M = 31.7532; free.p_M = 1; units.p_M = 'J/d.cm^3'; label.p_M = '[p_M], vol-spec somatic maint';

par.p_T = 0; free.p_T = 0; units.p_T = 'J/d.cm^2'; label.p_T = '{p_T}, surf-spec somatic maint';

par.k_J = 0.002; free.k_J = 0; units.k_J = '1/d'; label.k_J = 'maturity maint rate coefficient';

par.E_G = 5217.2023; free.E_G = 1; units.E_G = 'J/cm^3'; label.E_G = '[E_G], spec cost for structure';

par.E_Hb = 2.228e+01; free.E_Hb = 1; units.E_Hb = 'J'; label.E_Hb = 'maturity at birth';

par.E_Hj = 4.574e+01; free.E_Hj = 1; units.E_Hj = 'J'; label.E_Hj = 'maturity at metam';

par.E_Hp = 4.520e+05; free.E_Hp = 1; units.E_Hp = 'J'; label.E_Hp = 'maturity at puberty';

par.h_a = 4.253e-08; free.h_a = 1; units.h_a = '1/d^2'; label.h_a = 'maturity at puberty';

par.s_G = 0.0001; free.s_G = 0; units.s_G = '-'; label.s_G = 'Gompertz stress coefficient';

%% other parameters

par.del_M = 0.1116; free.del_M = 1; units.del_M = '-'; label.del_M = 'shape coefficient for total length';

par.del_Me = 0.065987; free.del_Me = 1; units.del_Me = '-'; label.del_Me = 'shape coefficient for embryo';

par.del_Ms = 0.042345; free.del_Ms = 1; units.del_Ms = '-'; label.del_Ms = 'shape coefficient for standard length';

par.f = 1; free.f = 0; units.f = '-'; label.f = 'scaled functional response for 0-var data';

par.f_F = 0.47343; free.f_F = 1; units.f_F = '-'; label.f_F = 'scaled functional response for GSI data';

par.f_LW = 0.71141; free.f_LW = 1; units.f_LW = '-'; label.f_LW = 'scaled functional response for LW data';

par.f_LWN = 0.82752; free.f_LWN = 1; units.f_LWN = '-'; label.f_LWN = 'scaled functional response for LWN data';

par.f_tL = 0.71729; free.f_tL = 1; units.f_tL = '-'; label.f_tL = 'scaled functional response for tL_H data';

par.f_tWw = 1.1575; free.f_tWw = 1; units.f_tWw = '-'; label.f_tWw = 'scaled functional response for tWw data';

par.b_H = 100018050.1691; free.b_H = 1; units.b_H = '1/d'; label.b_H = 'rate of reproductive reserve ripeness';

%% set chemical parameters from Kooy2010

[par, units, label, free] = addchem(par, units, label, free, metaData.phylum, metaData.class);

%% Pack output:

txtPar.units = units; txtPar.label = label; par.free = free;

Supplementary Data 6- Matlab code for “pars_init” file for parasitism simulation model

Function [par, metaPar, txtPar] = pars_init_Salvelinus_namaycush_sisc_repro(metaData)

metaPar.model = 'abj';

%% reference parameter (not to be changed)

par.T_ref = 293.15; free.T_ref = 0; units.T_ref = 'K'; label.T_ref = 'Reference temperature';

%% core primary parameters

par.T_A = 8000; free.T_A = 0; units.T_A = 'K'; label.T_A = 'Arrhenius temp';

par.z = 11.9994; free.z = 1; units.z = '-'; label.z = 'zoom factor';

par.F_m = 6.5; free.F_m = 0; units.F_m = 'l/d.cm^2'; label.F_m = '{F_m}, max spec searching rate';

par.kap_X = 0.8; free.kap_X = 0; units.kap_X = '-'; label.kap_X = 'digestion efficiency of food to reserve';

par.kap_P = 0.1; free.kap_P = 0; units.kap_P = '-'; label.kap_P = 'faecation efficiency of food to faeces';

par.v = 0.016443; free.v = 1; units.v = 'cm/d'; label.v = 'energy conductance';

par.kap = 0.60285; free.kap = 1; units.kap = '-'; label.kap = 'allocation fraction to soma';

par.kap_R = 0.95; free.kap_R = 0; units.kap_R = '-'; label.kap_R = 'reproduction efficiency';

par.p_M = 31.7532; free.p_M = 1; units.p_M = 'J/d.cm^3'; label.p_M = '[p_M], vol-spec somatic maint';

par.p_T = 0; free.p_T = 0; units.p_T = 'J/d.cm^2'; label.p_T = '{p_T}, surf-spec somatic maint';

par.k_J = 0.002; free.k_J = 0; units.k_J = '1/d'; label.k_J = 'maturity maint rate coefficient';

par.E_G = 5217.2023; free.E_G = 1; units.E_G = 'J/cm^3'; label.E_G = '[E_G], spec cost for structure';

par.E_Hb = 2.228e+01; free.E_Hb = 1; units.E_Hb = 'J'; label.E_Hb = 'maturity at birth';

par.E_Hj = 4.574e+01; free.E_Hj = 1; units.E_Hj = 'J'; label.E_Hj = 'maturity at metam';

par.E_Hp = 4.520e+05; free.E_Hp = 1; units.E_Hp = 'J'; label.E_Hp = 'maturity at puberty';

par.h_a = 4.253e-08; free.h_a = 1; units.h_a = '1/d^2'; label.h_a = 'maturity at puberty';

par.s_G = 0.0001; free.s_G = 0; units.s_G = '-'; label.s_G = 'Gompertz stress coefficient';

%% other parameters

par.b_H = 100018050.1691; free.b_H = 1; units.b_H = '1/d'; label.b_H = 'rate of reprductive reserve ripeness';

par.del_M = 0.1116; free.del_M = 1; units.del_M = '-'; label.del_M = 'shape coefficient for total length';

par.del_Me = 0.065987; free.del_Me = 1; units.del_Me = '-'; label.del_Me = 'shape coefficient for embryo';

par.del_Ms = 0.042345; free.del_Ms = 1; units.del_Ms = '-'; label.del_Ms = 'shape coefficient for standard length';

par.f = 1; free.f = 0; units.f = '-'; label.f = 'scaled functional response for 0-var data';

par.f_F = 0.47343; free.f_F = 1; units.f_F = '-'; label.f_F = 'scaled functional response for GSI data';

par.f_LW = 0.71141; free.f_LW = 1; units.f_LW = '-'; label.f_LW = 'scaled functional response for LW data';

par.f_LWN = 0.82752; free.f_LWN = 1; units.f_LWN = '-'; label.f_LWN = 'scaled functional response for LWN data';

par.f_tL = 0.71729; free.f_tL = 1; units.f_tL = '-'; label.f_tL = 'scaled functional response for tL_H data';

par.f_tWw = 1.1575; free.f_tWw = 1; units.f_tWw = '-'; label.f_tWw = 'scaled functional response for tWw data';

par.s_m = 0.75; free.s_m = 0; units.s_m = '-'; label.s_m = 'stress factor due to parasitism';

par.s_v = 0.07; free.s_v = 0; units.s_v = '-'; label.s_v = 'stress factor on v due to lipid';

par.s_kr = 2; free.s_kr = 0; units.s_kr = '-'; label.s_kr = 'stress factor on kap_R due to parasitism';

%% set chemical parameters from Kooy2010

[par, units, label, free] = addchem(par, units, label, free, metaData.phylum, metaData.class);

%% Pack output:

txtPar.units = units; txtPar.label = label; par.free = free;

Supplementary Data 7- Matlab code for “predict” file for base model

function [prdData, info] = predict_Salvelinus_namaycush_sisc(par, data, auxData)

% unpack par, data, auxData

cPar = parscomp_st(par); vars_pull(par);

vars_pull(cPar); vars_pull(data); vars_pull(auxData);

%filter check -- simply doesn't let scaled functional response go out of

%bounds

filterChecks = f_LW <0 || f_tL <0 || f_F <0 || f_tWw <0 || f_LWN <0 ;

if filterChecks

info = 0;

prdData = {};

return;

end

%% compute temperature correction factors

TC = tempcorr(temp.am, T_ref, T_A);

TC_ab = tempcorr(temp.ab, T_ref, T_A);

TC_ap = tempcorr(temp.ap, T_ref, T_A);

TC_am = tempcorr(temp.am, T_ref, T_A);

TC_LN = tempcorr(temp.LN, T_ref, T_A);

TC_tWw = tempcorr(temp.tWw, T_ref, T_A);

TC_WwN = tempcorr(temp.WwN, T_ref, T_A);

% life cycle

pars_tj = [g k l_T v_Hb v_Hj v_Hp];

[t_j, t_p, t_b, l_j, l_p, l_b, l_i, rho_j, rho_B, info] = get_tj(pars_tj, f);

s_M = l_j/l_b; % acceleration factor

% initial

pars_UE0 = [V_Hb; g; k_J; k_M; v]; % compose parameter vector

U_E0 = initial_scaled_reserve(f, pars_UE0);

E_0 = p_Am * U_E0; % J, initial energy in egg

Ww_0 = E_0 * w_E/ mu_E/ d_E; % g, egg wet weight

% birth

L_b = L_m * l_b; % cm, structural length at birth of foetus at f = 1

Lw_b = L_b/ del_Me; % cm, total length at birth at f

aT_b = t_b/ k_M/ TC_ab; % d, age at birth at f and T stolen from greenback cutthroat

% metamorphosis

L_j = L_m * l_j; % cm, structural length at birth of foetus at f = 1

Lw_j = L_j/ del_Ms; % cm, total length at metamorphosis at f

% puberty

L_p = L_m * l_p; % cm, structural length at puberty at f

Lw_p = L_p/ del_M; % cm, total length at puberty at f

Ww_p = L_p^3 *(1 + f * w); % g, wet weight at puberty stolen from greenback cutthroat trout

aT_p = aT_b + (t_p - t_b)/ k_M/ TC_ap; % d, age at puberty at f and T

% ultimate

L_i = L_m * l_i; % cm, ultimate structural length at f

Lw_i = L_i/ del_M; % cm, ultimate total length at f

Ww_i = L_i^3 * (1 + f * w); % g, ultimate wet weight

% life span

pars_tm = [g; l_T; h_a/ k_M^2; s_G]; % compose parameter vector at T_ref

t_m = get_tm_s(pars_tm, f, l_b); % -, scaled mean life span at T_ref

aT_m = t_m/ k_M/ TC_am; % d, mean life span at T

% pack to output

prdData.ab = aT_b;

prdData.ap = aT_p;

prdData.am = aT_m;

prdData.Lb = Lw_b;

prdData.Lj = Lw_j;

prdData.Lp = Lw_p;

prdData.Li = Lw_i;

prdData.Ww0 = Ww_0;

prdData.Wwp = Ww_p;

prdData.Wwi = Ww_i;

%% uni-variate data

% time - Length

[t_j, t_p, t_b, l_j, l_p, l_b, l_i, rho_j, rho_B] = get_tj(pars_tj, f_tL);

kT_M = TC * k_M; rT_B = rho_B * kT_M; rT_j = rho_j * kT_M; tT_j = (t_j - t_b)/ kT_M;

L_b = L_m * l_b; L_j = L_m * l_j; L_i = L_m * l_i;

L_bj = L_b * exp(tL_H(tL_H(:,1) < tT_j,1) * rT_j/ 3);

L_ji = L_i - (L_i - L_j) * exp( - rT_B * (tL_H(tL_H(:,1) >= tT_j,1) - tT_j)); % cm, expected length at time

ELw_H = [L_bj; L_ji]/ del_M;

% time - wet Weight

[t_j, t_p, t_b, l_j, l_p, l_b, l_i, rho_j, rho_B] = get_tj(pars_tj, f_tWw);

kT_M = TC_tWw * k_M; rT_B = rho_B * kT_M; rT_j = rho_j * kT_M; tT_j = (t_j - t_b)/ kT_M;

L_b = L_m * l_b; L_j = L_m * l_j; L_i = L_m * l_i;

L_bj = L_b * exp(tWw(tWw(:,1) < tT_j,1) * rT_j/ 3);

L_ji = L_i - (L_i - L_j) * exp( - rT_B * (tWw(tWw(:,1) >= tT_j,1) - tT_j)); % cm, expected length at time

L = [L_bj; L_ji];

EWw = L.^3 * (1 + f_tWw * w);

% length-weight

EWw_L = (LW(:,1) * del_M).^3 * (1 + f_LW * w);

% length - number of offspring

pars_R = [kap; kap_R; g; k_J; k_M; L_T; v; U_Hb; U_Hj; U_Hp]; % compose parameter vector

EN = 365 * TC_LN * reprod_rate_j(LN(:,1) * del_M, f_LWN, pars_R); % #, number of offspring per year

% wet weight - fecundity

ENWw = 365 * TC_WwN * reprod_rate_j((WwN(:,1)/ (1 + f_LWN * w)).^(1/3), f_LWN, pars_R); % #, fecundity

% growth - reproduction

%% No Parasitism, No skipped spawning

V_0 = (length0.tL_1 * del_M)^3; % cm^3, inital structure

M_E0 = f_F * m_Em * M_V * V_0; % mol; mass of reserve

SV0 = [M_V*V_0, M_E0, 0, 0];

tinterval = linspace (0, 365, 500); % integrate over a year

[time, OUT] = ode23s(@dget_VEROv, tinterval, SV0, [], par, f_F, temp.tWw_1, signal.tWw_1, s_M);

%

EL_aux = ((OUT(:,1)/M_V).^(1/3))/del_M; % cm, physical length

EWw_soma_aux = (w_V * OUT(:,1)/d_V + w_E * (OUT(:,2) + OUT(:,3))/d_E); % g, wet weight (M_V + M_E + M_R) excludes ovarian mass

M_ovaries_aux = w_E * OUT(:,4)/d_E; % g, wet mass of ovaries, ripe reproductive reserve

Ww_total = EWw_soma_aux + M_ovaries_aux; % g, wet weight (M_V + M_E + M_R) excludes ovarian mass

%

EL_1 = interp1(time, EL_aux, tL_1(:,1), 'spline'); % cm, physical length; returns the values of predictions at tL_1(:,1)

EWw_1 = interp1(time, Ww_total, tWw_1(:,1), 'spline'); % g, (total) wet weight; returns the values of predictions at tWw_(:,1)

%

M_ovaries_1 = interp1(time, M_ovaries_aux, 365, 'spline'); % g, wet mass of ovaries, returns the values of predictions at day 365

%% pack to output

prdData.tL_H = ELw_H;

prdData.tWw = EWw;

prdData.LW = EWw_L;

prdData.LN = EN;

prdData.WwN = ENWw;

prdData.tL_1 = EL_1;

prdData.tWw_1 = EWw_1;

prdData.tMov_1 = M_ovaries_1;

end

%%

function dVEROv = dget_VEROv(t, SV, par, f_W, Temp, tE2, s_M)

% t: scalar with age

% SV: 4-vector with the state variables

% M_V (mass of structure), m_E (scaled reserve density),

% M_R (reproductive mass), M_Ov (mass in gametes)

% dVEROv: 4-vector with (dM_V/dt, dM_E/dt, dM_R/dt,dM_Ov/dt)

Mass_V = SV(1); % mol, structural mass

M_E = SV(2); % mol, mass of reserve

M_R = SV(3); % mol, mass investment to reproduction

M_OV = SV(4); % mol, mass of ovaries

m_E = M_E/Mass_V; % mol/mol, reserve density

cPar = parscomp_st(par); % compute compound parameters

vars_pull(par); vars_pull(cPar); % unpack par, cPar

TC = tempcorr(Temp, T_ref, T_A);

L = (Mass_V/M_V)^(1/3); % cm, Structural volumetric length

L2 = L*L;

% forcing/signal

% interpolate the E2 data

W = (w_V * Mass_V + w_E * m_E.* Mass_V)/d_V; % g, wet weight

Vpl = 2.86 * W/100; % ml, plasma volume

M_E2 = tE2(:,2) * Vpl; % mol, E2

m_H = (t>100)*(t<=365) * interp1(tE2(:,1), M_E2,t,'pchip')/Mass_V; % mol E2/mol V, estradiol density

j_VtG = (M_R>0)* TC * b_H * m_H *M_R/Mass_V; % mol ER/molV.time

j_EAm = s_M * J_E_Am / Mass_V^(1/3) / M_V^(2/3);

J_EA = s_M * J_E_Am * f_W * L2;

if kap * j_EAm * m_E/m_Em >= j_E_M

r = TC * (j_EAm * m_E/m_Em - j_E_M / kap)/(m_E +y_E_V/kap); % 1/d, specific growth rate

J_EC = m_E * Mass_V * (TC * s_M * v / L - r) ; % mol/d, reserve mobilization rate

J_R =(1 - kap) * J_EC - TC*k_J * M_Hp - j_VtG * Mass_V; % mol/d, reproduction rate

J_OV = kap_R * j_VtG * Mass_V ; % mol/d, VtG production rate

end

%

if kap * j_EAm * m_E/m_Em < j_E_M && ( M_OV > 0 || M_R>0)

r = 0; % d^-1, specific growth rate

a_maint = (t<=100) * 1 + (t>100) * M_R/(M_R+M_OV);

J_EC =m_E * Mass_V * (TC* s_M * v / L - r); % mol/d, reserve mobilization rate

Js_maint = TC * J_E_M * Mass_V/M_V - kap * J_EC; % somatic maintenance that is not covered by the kappa-branch

J_R = (1 - kap) * J_EC - TC*k_J * M_Hp - a_maint * Js_maint - j_VtG * Mass_V; % mol/d, reproduction flux

J_OV = kap_R * j_VtG * Mass_V - (1 - a_maint)* Js_maint; % mol/d, VtG production rate

end

%

if kap * j_EAm * m_E/m_Em < j_E_M && M_OV <= 0 && M_R <= 0

% shrinking

r = TC * (j_EAm * m_E/m_Em - j_E_M / kap)/(m_E + kap_G * y_E_V/kap); % d^-1, specific growth rate

J_EC =m_E * Mass_V * (TC* s_M * v / L - r); % mol/d, reserve mobilization rate

J_R = (1 - kap) * J_EC - TC*k_J * M_Hp - j_VtG * Mass_V; % mol/d, reproduction flux

J_OV = kap_R * j_VtG * Mass_V; % mol/d, VtG production rate

end

dM_E = J_EA - J_EC; % mol/d, change in energy in reserve

dM_V = r * Mass_V; % mol/d, change in structural mass

dM_R = J_R; % mol/d, change in reproductive reserve

dM_OV = J_OV; % mol/d, change in ovarian mass

dVEROv = [dM_V; dM_E; dM_R; dM_OV];

end

%% Files used for the estimation of parameters for the siscowet lake charr

% (Salvelinus namaycush). They use functions in DEBtool package

% (https://github.com/add-my-pet/DEBtool_M)

%

% THE CONSEQUENCES OF SEA LAMPREY PARASITISM ON LAKE TROUT ENERGY BUDGETS

% Tyler J. Firkus, Konstadia Lika, Noah Dean, Cheryl A. Murphy

close all

global pets

pets = {'Salvelinus_namaycush_sisc'};

check_my_pet(pets);

estim_options('default');

estim_options('max_step_number', 500);

estim_options('max_fun_evals',5e3);

estim_options('pars_init_method', 2);

estim_options('results_output', 2);

estim_options('method', 'no');

estim_pars;

Supplementary Data 8- Matlab code for “predict” file for parasitism simulation model

%% Files used for simulating the effects of parasitism on siscowet lake charr

% (Salvelinus namaycush). They use functions in DEBtool package

% (https://github.com/add-my-pet/DEBtool_M)

%

% THE CONSEQUENCES OF SEA LAMPREY PARASITISM ON LAKE TROUT ENERGY BUDGETS

% Tyler J. Firkus, Konstadia Lika, Noah Dean, Cheryl A. Murphy

close all; clear all;

[data, auxData, metaData, txtData, weights] = mydata_Salvelinus_namaycush_sisc_simu;

[par, metaPar, txtPar] = pars_init_Salvelinus_namaycush_sisc_simu(metaData);

[prdData, time_1, time_2, time_3, time_4, time_5, time_6, OUT_mR_1, M_ovaries_aux_1, ...

OUT_mR_2, M_ovaries_aux_2, OUT_mR_3, M_ovaries_aux_3, OUT_mR_4, M_ovaries_aux_4, ...

OUT_mR_5, M_ovaries_aux_5, OUT_mR_6, M_ovaries_aux_6, info] = ...

predict_Salvelinus_namaycush_sisc_simu(par, data, auxData);

figure(1)

hold on

plot(time_1, OUT_mR_1, 'b', time_1, OUT_mR_2, 'k', time_1, OUT_mR_3, 'r', time_1, OUT_mR_4, 'b:', time_1, OUT_mR_5, 'k:', time_1, OUT_mR_6, 'r:')

xlabel('time, d')

ylabel('wet weight of reproductive buffer, g')

figure(2)

hold on

plot(time_1, M_ovaries_aux_1,'b', time_2, M_ovaries_aux_2,'k', time_3, M_ovaries_aux_3,'r', time_4, M_ovaries_aux_4, 'b:', time_5, M_ovaries_aux_5, 'k:', time_6, M_ovaries_aux_6, 'r:')

xlabel('time, d')

ylabel('wet weight of ovaries, g')

figure(3)

hold on

plot(time_1, prdData.tL_1,'b', time_2, prdData.tL_2,'k', time_3, prdData.tL_3,'r', time_4, prdData.tL_4, 'b:', time_5, prdData.tL_5, 'k:', time_6, prdData.tL_6, 'r:')

xlabel('time, d')

ylabel('physical length, cm')

t_span = linspace(0,365,500)';

signal = interp1(auxData.signal.tWw_1(:,1), auxData.signal.tWw_1(:,2),t_span,'pchi');

figure(4)

hold on

plot(t_span, 1e9*signal, auxData.signal.tWw_1(:,1), 1e9*auxData.signal.tWw_1(:,2),'ok'), hold on

xlabel('time, d')

ylabel('E2, nmol/ml')

set(gca, 'FontSize', 15, 'Box', 'on')
